# Identification of New Ciliary Signaling Pathways in the Brain and Insights into Neurological Disorders

**DOI:** 10.1523/JNEUROSCI.0800-24.2025

**Published:** 2025-07-07

**Authors:** Abdelhalim Loukil, Emma Ebright, Karama Hamdi, Elizabeth Menzel, Akiyoshi Uezu, Yudong Gao, Scott H. Soderling, Sarah C. Goetz

**Affiliations:** ^1^Pediatrics and Rare Diseases Group, Sanford Research, Sioux Falls, South Dakota 57104; ^2^Department of Pediatrics, Sanford School of Medicine, University of South Dakota, Sioux Falls, South Dakota 57105; ^3^Department of Pharmacology and Cancer Biology, Duke University School of Medicine, Durham, North Carolina 27710; ^4^Department of Cell Biology, Duke University Medical School, Durham, North Carolina 27710

**Keywords:** iBioID, mature brain, neurodevelopmental disorders, neurons, primary cilia

## Abstract

Primary cilia are conserved sensory hubs essential for signaling transduction and embryonic development. Ciliary dysfunction causes a variety of developmental syndromes with neurological features and cognitive impairment whose basis mostly remains unknown. Despite connections to neural function, the primary cilium remains an overlooked organelle in the brain. Most neurons have a primary cilium; however, it is still unclear how this organelle modulates brain architecture and function, given the lack of any systemic dissection of neuronal ciliary signaling. Here, we present the first in vivo glance at the molecular composition of cilia in the mouse brain. We have adapted in vivo proximity-dependent biotin identification (iBioID), targeting the biotin ligase BioID2 to primary cilia in neurons of male and female mice. We identified tissue-specific signaling networks residing in neuronal cilia, including Eph/Ephrin signaling. We also uncovered a novel connection between primary cilia and gamma-aminobutyric acid signaling. Our iBioID ciliary network presents a wealth of new and neural-specific ciliary signaling proteins and yields new insights into neurological disorders. Our findings are a promising first step in defining the fundamentals of ciliary signaling and their roles in shaping neural circuits and behavior. In the future, this work can be extended to pathological conditions of the brain, with the goal of identifying ciliary signaling pathways disrupted in these disorders and the ultimate aim of finding novel therapeutic strategies.

## Significance Statement

Primary cilia are sensory hubs crucial for signal transduction and embryonic development. Mutations in ciliary genes can lead to developmental disorders characterized by a wide spectrum of neurological impairments, the molecular basis of which is unknown. Despite its importance, the cilium's functions in the brain remain poorly understood. In this manuscript, we have adapted the in vivo proximity-dependent biotin identification to identify the signaling outputs of cilia in neurons. We uncovered novel protein networks in neuronal cilia, including Eph/Ephrin and gamma-aminobutyric acid receptor pathways. We also generated the first ciliary protein network in neurons and shared a wealth of neural hits that can help uncover how cilia mediate neural function and can become perturbed in neurological disorders.

## Introduction

The primary cilium is a sensory organelle that is essential for embryonic development and the homeostasis of multiple tissues and organs. It protrudes from the cell to detect extracellular signals and generates specific downstream responses (Goetz and Anderson, 2010). Cilia therefore play critical roles in transducing major signaling pathways, including the sonic hedgehog (SHH) pathway (Haycraft et al., 2001, 2005; Taulman et al., 2001; Pazour et al., 2002; Huangfu et al., 2003; Goetz and Anderson, 2010; Goetz et al., 2012). Mutations in ciliary genes are associated with multiple recessive genetic conditions, collectively termed “ciliopathies,” which are often characterized by severe neurological deficits (Goetz and Anderson, 2010; Waters and Beales, 2011; Braun and Hildebrandt, 2017; Reiter and Leroux, 2017; Anvarian et al., 2019), including cognitive impairment, motor dysfunction, and brain malformations (Sattar and Gleeson, 2011; Valente et al., 2014; Guo et al., 2015; Youn and Han, 2018). Recent evidence has linked cilia dysfunction to additional neurodevelopmental and neurodegenerative disorders (Keryer et al., 2011; Valente et al., 2014; Kaliszewski et al., 2015; Bowie et al., 2018; Dhekne et al., 2018; Khan et al., 2021; Loukil et al., 2021; Sobu et al., 2021). While cilia are intimately linked to brain disorders, the ciliary signaling pathways underlying these diseases are unclear, as are the molecular mechanisms of cilia-driven pathogenicity in neurological disorders.

Robust evidence links ciliary signaling to the establishment and maintenance of neural connectivity and function (Han et al., 2008; Kumamoto et al., 2012; Tong et al., 2014; Guo et al., 2019; Bowie and Goetz, 2020; Sheu et al., 2022). In a previous study, our laboratory found that cilia ablation in the adult cerebellum caused the degeneration of Purkinje neurons and defective neuronal connectivity (Bowie and Goetz, 2020). Moreover, neuronal cilia are reported to form novel tetrapartite structures with pre- and postsynapses and astrocytes in which the cilium has access to synaptic activity in the surrounding environment (Wu et al., 2023). Consistent with these findings, axons were found to release serotonin directly onto neuronal cilia, triggering changes in nuclear actin and increased chromatin accessibility (Sheu et al., 2022). Despite such striking findings, the molecular networks within the neuronal cilium that mediate adult brain function and homeostasis are poorly defined. What signals do neuronal cilia detect and process? What are the downstream regulators and their effects on the neuron's function and structure? In the absence of such knowledge, identifying neuronal circuits that rely on neuronal cilia will likely remain difficult.

The cilium's sensory function is tightly linked to its protein composition, modulated by a precisely regulated trafficking of molecules in and out of the cilium by intraflagellar transport (IFT) and other associated ciliary membrane trafficking pathways (Kozminski et al., 1993; Pazour et al., 2000; Ye et al., 2018). Recent work has uncovered structural diversity among cilia of different cell types in the brain. For example, while neuronal cilia dock directly to the plasma membrane, astrocytic cilia are present in pockets or within the soma (Ott et al., 2023; Wu et al., 2023). These structural differences between cell types imply that cilia differ in their composition and regulation. Cilia use their intrinsic molecular composition to drive downstream signals and mediate the appropriate cellular response in a tissue-specific manner. The neuronal cilium harbors a number of specialized signaling molecules specifically expressed in neural tissues, including somatostatin receptor 3 (SSTR3), serotonin receptor 5-HT6, and adenylyl cyclase 3 (ADCY3; Hamon et al., 1999; Händel et al., 1999; Brailov et al., 2000; Schulz et al., 2000; Bishop et al., 2007; Berbari et al., 2008a,b; Guadiana et al., 2016). However, the identification of the cilia proteome in neurons and how it may modulate signaling networks and neural circuitry is still lacking. To date, mammalian ciliary proteomes have been defined mainly through the use of immortalized cell lines and thus do not account for tissue specificity or the in vivo microenvironment. This is particularly true for neuronal cilia, which are present within a neural tissue with complex organization and a variety of cellular subtypes. Recent transmission electron microscopy studies underscore the involvement of neuronal cilia in neural circuitry (Ott et al., 2023; Wu et al., 2023), which can't be recapitulated easily in vitro.

In order to address this gap in knowledge, we applied an in vivo proximity-dependent biotin identification (iBioID) strategy (Kim et al., 2016; Uezu et al., 2016) to capture a first glimpse of the proteome of neuronal cilia in the mouse brain, optimizing the experimental conditions that allowed the efficient purification of ciliary proteins from the brain tissue. Quantitative mass spectrometry uncovered 389 proteins potentially localizing within neuronal cilia, including 42 known ciliary proteins. Gene ontology (GO) analysis revealed that at least 202 genes were associated with or mutated in rare neurological disorders. Using this approach, we have identified novel ciliary signaling nodes in the brain that were not revealed in prior studies of the ciliary proteomes of immortalized cell lines. Based on these findings, we conclude that cilia in neurons are highly populated with neuropeptide and neuroendocrine receptors, ion channels, and calcium-binding proteins. We also identified several molecular modules involved in cell–cell communication, including axon guidance and the Eph/Ephrin pathway. Our findings unveil a functional linkage between primary cilia and regulation of the ephrin receptor, EPHA4, in the mature brain. This work presents a powerful new tool for identifying the in vivo proteomes of primary cilia in neurons in the brain.

## Materials and Methods

### Animals and tamoxifen injections

FVB/NJ mice (stock number. 001800) and Rosa26-LSL-Cas9 knockin (stock number 026175) were obtained from Jackson Laboratory. Transgenic mice expressing Arl13b-mCherry were a gift from Dr. Kathryn Anderson. We also used Ttbk2tm1a(EUCOMM)Hmgu (Ttbk2^fl^ allele, International Mouse Strain Resource) and Ubc-Cre-ERT2 (Jackson Laboratory, strain #007001) mouse lines. Corn oil or tamoxifen injections (100 µl of 20 mg/ml) were performed intraperitoneally once a day for 5 d. Seven male mice were then killed, and brains were collected 6 weeks (+1 d) postinjections. Mice were housed (up to five mice per cage) in the Division of Laboratory Animal Resources facility at Duke University. All the experimental procedures have been approved by the Duke Institutional Animal Care and Use Committee and followed the National Institutes of Health guidelines.

### Cloning and IMCD3 culture

cDNAs for mouse GPCRs, SSTR3, or MCHR1 cDNAs were cloned into the pAAV-BirA2-HA-linker-BirA2-HA (gift from Dr. Scott Soderling, Duke University). BirA2-HA was C-terminally fused to either SSTR3 or MCHR1. Similarly, BirA was fused to NPHP3 (a.a 1-203) fragment. Plasmids containing gene sequences for EphA4 (pDONR223-EPHA4, Addgene, 23919) and EphB3 (FUW-ubiquitin-EphB3-SV40-GFP, Addgene, 65443), two ciliary proteome candidates, were obtained and used to perform gateway recombination cloning. pDONR223-EPHA4 was directly cloned into PFLAP-DEST using the LR Clonase reaction (Thermo Fisher Scientific, 11791020). FUW-ubiquitin-EphB3-SV40-GFP underwent PCR amplification and purification using the following primer sequences to amplify the EphB3 sequence: forward 5′CACCATGGCCAGAGCCCGCC 3′, reverse 5′ TCAGACCTGCACAGGCAGCGT 3′. With the PCR purified product, TOPO cloning was performed (Thermo Fisher Scientific, K240020). Then, pENTR/D-TOPO-EphB3 was cloned into pcDNA3.1/nV5-DEST (Thermo Fisher Scientific, 12290010). After amplification of the final cloned constructs, transient transfection was performed in mouse inner medullary collecting duct cells (mIMCD-3; ATCC, CRL-2123). These were plated on 15 mm coverslips and maintained in DMEM: F-12, HEPES (Thermo Fisher Scientific, 11330057) supplemented with 10% v/v heat-inactivated fetal bovine serum (Thermo Fisher Scientific, 10082147). Transient transfection was performed accordingly with Lipofectamine 2000 Transfection Reagent (Thermo Fisher Scientific, 11668019).

### AAV production and purification

For each adeno-associated virus (AAV), we transfected six 15 cm plates of HEK293T cells with pAd-DeltaF6, pAAV serotype 2/9, and pAAV-GPCR-BirA2-HA or pAAV-BirA2-HA. HEK293T cells were cultured in DMEM with 10% fetal bovine serum. We used 7.5 mM polyethylenimine and incubated transfection complexes for 18 h. Growth media were replaced the next day. After 24 h, cells and supernatants are collected and centrifuged at room temperature for 5 min at 12,000 rpm. Pellets were then resuspended in 4 ml of cell lysis buffer (15 mM NaCl, 5 mM Tris–HCl), pH 8.5. We performed three cycles of freeze–thawing in a dry ice–ethanol bath and a 37°C water bath. Benzonase (50 U/ml) was added to cell lysates and incubated for 30 min in a 37°C water bath. Lysates were centrifuged at 4,500 rpm for 30 min at 4°C. Supernatants were collected and added on a gradient of 15, 25, 40, and 60% of iodixanol solution. We centrifuged at 30,000 rpm for 2 h to carefully collect the viral solution. We concentrated AAV viruses using Amicon filters and washed them four times with 1× phosphate-buffered saline (PBS). We finally resuspended them in 100 μl. To inject equal amounts of viruses, we tittered viral solutions by quantitative PCR. Until needed, AAVs were stored at −80°C.

### iBioID of neuronal cilia

#### AAV intracranial injections in P0–P2 pups

AAV viruses coding for BirA2, MCHR1-BirA2, or SSTR3-BirA2 were produced, purified, and titrated. Intracranial injections of AAVs were performed in Postnatal Day (P)0–P2 brain pups. Note that wild-type FVB/NJ background was used for the quantitative proteomics and AC mice for immunohistochemical labeling. For optimal labeling throughout the forebrain, we injected ice-anesthetized pups with 1.5–2.5 μl per hemisphere of concentrated AAVs diluted in 1× PBS (from 10^10^ to 1.5 10^11^ virus/μl) using a 10 μl Hamilton syringe and a 1 in needle (33 gauge needle with a 20° bevel). A heating pad was used to facilitate the recovery of injected pups until their movement pattern and skin color were back to normal. After injections, the whole litter was reintroduced to the dam and closely monitored for at least 24 h.

#### Biotin labeling in mice

At P19–P25, we subcutaneously injected 1 ml of 6.66 μM of biotin diluted in 1× PBS (sterile) once a day for 5 d. We continued subcutaneous injections that we combined with the intraperitoneal injections (1 ml per day) for 6 d. The two injections were performed at different times during the day for the welfare of the animal. AAV-BirA2-, MCHR1-BirA2-, and SSTR3-BirA2-injected brains were carefully extracted at P31–P36 of age. The cerebellum that showed weak biotin labeling was removed. The rest of the brain was snap-frozen in liquid nitrogen until pulldowns were performed.

#### Pulldowns of biotinylated protein from brains

A critical note: Throughout the purification step, personal protective equipment, including a mask, a clean coat, gloves, and a bouffant cap, should be worn to diminish human keratin contamination. All solutions were also freshly prepared in similar conditions. The quantitative proteomics experiment was performed with AAV injections of 10 different litters on different days. For each biological replicate, we used 6–7 brains per condition (a total of 61 mice, including 28 males and 33 females) that we pooled and crushed into fine powder with a mortar and pestle dipped in liquid nitrogen. The lysis buffer was freshly prepared. Its composition is as follows: [1% NP-40 (85124, Thermo Fisher Scientific), two protease inhibitor tablets (Roche), 20 μM MG132 (Sigma-Aldrich), 50 mM beta-glycerol phosphate (Sigma-Aldrich), and 1:1,000 benzonase nuclease (250 U/μl) from Sigma-Aldrich]. Brain powder was put into 10 ml of lysis buffer in Corning 15 ml tubes on ice for each condition. We incubated the extracts on ice for 30 min to help lyse the brain powder, followed by a sonication step: eight times for 3 s with an amplitude of 25%. We then added 1 ml of 10% SDS and incubated for 15 min on ice. Extracts were transferred to 1.5 ml low-retention tubes or ultracentrifuge tubes and incubated for an additional 15 min on ice. Lysates were then centrifuged at 15,000 rpm for 45 min at 4°C, and the supernatants were transferred to new 15 ml tubes (Corning). We next washed the NeutrAvidin beads five times with lysis buffer with additives. We added 120 μl of washed NeutrAvidin beads to each supernatant and incubated each pulldown at 4°C with tube rotation at a speed of 25 rpm. The next day, we centrifuged samples at 3,000 rpm for 3 min to recover the beads and remove the supernatant. The final step consisted of several washes sequentially performed on beads. We carried out three washes with 12 ml of each of the following solutions: (1) 2% SDS; (2) 1% Triton X-100, 1% deoxycholate, and 25 mM LiCl; and (3) 1 M NaCl (Uezu et al., 2016). For each wash, we rotated samples for 5 min at room temperature with a 30 rpm rotation speed. We then spun them at 3,000 rpm for 1 min after each wash. Next, we washed them four times with 50 mM ammonium bicarbonate. Beads were transferred to low-retention 1.5 ml tubes. A 220 μl of 2× Laemmli supplemented with 5 mM biotin was added, and samples were incubated for 8.5 min at 95°C. To remove any residual beads, we centrifuged them twice and recovered about 160 μl from each pulldown. We kept 20 μl to validate the level of biotin labeling for each sample by Western blot (WB). Samples were stored at −80°C until submitted for sequencing with mass spectrometry. We carried out WBs to assess the overall biotin labeling and a ciliary marker, ARL13B.

### Quantitative proteomics

#### Sample preparation

The Duke Proteomics Core Facility received nine samples (three replicates of BirA2, three of MCHR1, and three of SSTR3). Samples were supplemented with 160 μl of 10% SDS in 50 mM TEAB and then reduced with 10 mM dithiolthreitol for 30 min at 80°C and alkylated with 20 mM iodoacetamide for 30 min at room temperature. Next, they were supplemented with a final concentration of 1.2% phosphoric acid and 2,730 μl of S-Trap (ProtiFi) binding buffer (90% MeOH/100 mM TEAB). Proteins were trapped on the S-Trap, digested using 20 ng/μl sequencing grade trypsin (Promega) for 1 h at 47°C and eluted using 50 mM TEAB, followed by 0.2% FA and lastly using 50% ACN/0.2% FA. All samples were then lyophilized to dryness and resuspended in 12 μl 1%TFA/2% acetonitrile containing 12.5 fmol/μl yeast alcohol dehydrogenase (ADH_YEAST). From each sample, 3 μl was removed to create a QC pool sample which was run periodically throughout the acquisition period.

#### Quantitative analysis and methods

Quantitative liquid chromatography–tandem mass spectrometry (LC–MS/MS) was performed on 4 μl of each sample, using a nanoAcquity UPLC system (Waters) coupled to a Thermo Fusion Lumos high-resolution accurate mass tandem mass spectrometer (Thermo Fisher Scientific) via a nanoelectrospray ionization source. Briefly, the sample was first trapped on a Symmetry C18 20 mm × 180 μm trapping column (5 μl/min at 99.9/0.1 v/v water/acetonitrile), after which the analytical separation was performed using a 1.8 μm Acquity HSS T3 C18 75 μm × 250 mm column (Waters) with a 90 min linear gradient of 5–30% acetonitrile with 0.1% formic acid at a flow rate of 400 nanoliters/minute (nl/min) with a column temperature of 55°C. Data collection on the QExactive HF-X mass spectrometer was performed in a data-dependent acquisition mode of acquisition with a *r* = 120,000 (at m/z 200) full MS scan from m/z 375–1,500 with a target AGC value of 2 × 10^5^ ions followed by 30 MS/MS scans at *r* = 15,000 (at m/z 200) at a target AGC value of 5 × 10^4^ ions and 45 ms. A 20 s dynamic exclusion was employed to increase depth of coverage. The total analysis cycle time for each sample injection was ∼2 h.

Following 13 total UPLC-MS/MS analyses (excluding conditioning runs, but including four replicate QC injections), data were imported into Proteome Discoverer 2.2 (Thermo Fisher Scientific), and analyses were aligned based on the accurate mass and retention time of detected ions (“features”) using Minora Feature Detector algorithm in Proteome Discoverer. Relative peptide abundance was calculated based on area under the curve of the selected ion chromatograms of the aligned features across all runs. The MS/MS data were searched against the SwissProt *Mus musculus* database (downloaded in April 2018) with additional proteins, including yeast ADH1, bovine serum albumin, as well as an equal number of reversed-sequence “decoys” for false discovery rate determination. Mascot Distiller and Mascot Server (v 2.5, Matrix Sciences) were utilized to produce fragment ion spectra and to perform the database searches. Database search parameters included fixed modification on Cys (carbamidomethyl) and variable modifications on Met (oxidation) and Asn and Gln (deamidation). Peptide Validator and Protein FDR Validator nodes in Proteome Discoverer were used to annotate the data at a maximum 1% protein false discovery rate.

As an initial statistical analysis, we calculated fold changes between groups based on the median fold change (MCHR1 vs BirA2; SSTR3 vs BirA2). In addition, we performed a two-tailed heteroscedastic *t* test on log2-transformed data for each of these comparisons. We used the data to identify proteins that were specific to each of the comparisons. Briefly, proteins were filtered to include those with a twofold greater expression and a *p* value of <0.05. This resulted in 605 and 525 proteins for MCHR1 and SSTR3 comparisons, respectively.

### GO and cluster analysis

GO analysis was performed using Ingenuity Pathway Analysis (IPA). We followed the manufacturer's guidelines to extract the enriched molecular functions, enriched signaling pathways, and associations with diseases. Cytoscape 3 was employed to reconstitute the molecular networks of our cilia iBioID hits. We used the STRING app, in which we selected a full-string network with a confidence score cutoff of 0.3. Once the network is formed, we display the fold change as the size of the spheres and the *p* values with a colored continuous mapping. To highlight certain proteins, we uploaded a data table file as node table columns that we matched with the display name (case-insensitive). We then selected these hits and filled the spheres with the appropriate colors.

### Brain lysates

The brains collected from mice were ground with a pestle using liquid nitrogen and placed directly in dry ice. We then prepare the protein extraction reagent by adding one mini tablet of the Protease and Phosphatase Inhibitor Mini Tablets (catalog number A32961) to 10 ml of the T-PER Tissue Protein Extraction Reagent (Thermo Fisher Scientific, product number 78510/use a ratio of ∼1 g of tissue to 20 ml T-PER Reagent.). To 0.05 g from each tissue sample, we added 1 ml of the reagent T-PER with protease and phosphatase inhibitors and then homogenized well by pipetting up and down several times. The mixtures were centrifuged at 10,000 × *g* for 10 min, and the supernatants were transferred to new tubes. Before running an SDS gel, we took 20 µl of the lysate from each sample, mixed it with 5 µl of Laemmli buffer (4×), and heated it for 10 min at 95°C.

### WB analysis

Lysates were incubated at 95°C for 5 min. Denatured proteins were separated by SDS-PAGE and transferred onto a PVDF membrane. The membrane was saturated in 5% milk PBST (5% dry powdered milk, 0.1% Tween 20, 1× PBS) for 1 h and then incubated with primary antibodies overnight and secondary antibodies (peroxidase-conjugated affinipure or IRDye secondary antibodies). Membranes were then developed with an ECL reagent kit (BioRad Laboratories) or Li-COR imaging system. The WBs were quantified using the Fiji software.

### Primary neuronal culture

Primary neuronal cultures were derived from Cas9 knockin P0 pups. All brain dissections were performed in cold 1× PBS. After brain extraction, we carefully removed and discarded the meninges. Cortices and/or hippocampi were isolated and cut into pieces before being transferred to 15 cm tubes in 1× PBS. After discarding the PBS, we added a preheated dissociation solution (Mix A [40 ml 1× PBS, 10 mg DL-cysteine HCl (Sigma-Aldrich C9768), 10 mg BSA, 250 mg d-glucose (Sigma-Aldrich G6152)]; 5 U/ml papain; and 120 U/ml DNase). Dissociate for 30–40 min at 37°C. We then replaced the solution with neuronal media (50 ml neurobasal, 1 ml neuronal B27 supplement, 500 μl Glutamax, and 60 μl pen/strep). Mechanical dissociation is carefully performed using a 1 ml micropipette (10 times). Cells were plated on precoated poly-d-lysine (0.1 mg/ml, 37°C; 5% CO₂ for at least 2 h) coverslips in a 24-well plate. Cells were fixed at DIV10–15 with 4% paraformaldehyde for 15 min and permeabilized with cold 100% methanol for 5 min.

### IPSCs

To culture induced pluripotent stem cells (IPSCs), we incubated dishes with hESC-Qualified Matrigel (Thermo Fisher Scientific) diluted in DMEM/F12 media (Invitrogen) for 1 h at 37°C. Cells were cultured in mTeSR1 (STEMCELL Technologies). We used dispase (STEMCELL Technologies) to passage the cells. For cilia analysis, we plated Matrigel-coated iBidi slides with IPSCs and cultured them for 3 d in mTeSR1. Cells were then fixed with 4% paraformaldehyde (PFA) for 10 min and then permeabilized with cold methanol for 5 min.

### Immunofluorescence and microscopy

Neurons were fixed between DIV7 and DIV14 in 4% PFA/PBS (Santa Cruz Biotechnology, sc-281692) for 15 min at room temperature. Then, they were permeabilized with ice-cold methanol (VWR, BDH1135) for 5 min at −20°C. Neurons were blocked with blocking buffer PBS + 1% BSA and 0.2% Triton X-100 for 1 h at room temperature. Cells were fixed with 4%PFA for 5 min at room temperature and permeabilized with ice-cold methanol for 5 min at −20°C. Cells were blocked with blocking buffer PBS + 1% BSA, 0.2% Triton X-100, and 5% goat serum for 30 min at room temperature. Samples were then stained with their respective primary antibodies listed previously overnight at 4°C. The next morning, after washing with PBS three times for 5 min, samples were incubated with appropriate secondary antibodies for 1 h (neurons) or 30 min (transfected cells) at room temperature. Samples were then incubated with DAPI (Sigma-Aldrich, D9542, 1:1,000) and then washed with PBS three times for 5 min and mounted with ProLong Gold Antifade Mountant (Thermo Fisher Scientific, P36930). Mounted coverslips were then imaged on a Zeiss Axio Observer-Z1 widefield microscope equipped with a Zeiss Plan-Apochromat 63×/1.4 oil objective, an Axiocam 506 monochrome camera, and an Apotome setting. To accurately quantify the endogenous presence and overexpression of ciliary proteome candidates and ciliary markers, a Z-stack was acquired, a maximum intensity projection for each image was created, and the maximum intensity projections were analyzed using the Fiji software.

### Immunohistochemistry

Transcardiac perfusion of P31–P36 mice was performed using 1× PBS followed by 4% PFA. After extraction, the brain was fixed in 4% PFA for 2 h at 4°C. PFA was washed out with 1× PBS and replaced with 30% sucrose for overnight incubation. The brains were frozen in disposable embedding molds in OCT compound. We then generated coronal and sagittal brain sections of ∼20 μ, mounted on charged slides. To permeabilize the tissue, we incubate it with 1% Triton X-100 diluted in 1× PBS for 1 h. The tissue is blocked in blocking buffer [0.1% Triton X-100, 5% FBS (goat), 1% bovine serum albumin, 0.02% sodium azide, 2% FBS, 0.1% Triton X-100 in 1× PBS] for 1 h. Primary antibodies were incubated for 3 h at room temperature or overnight at 4°C, followed by 2 h of incubation with secondary antibodies (1:500; Alexa Fluor 488, Alexa Fluor 568, Alexa Fluor 647; Molecular Probes, Thermo Fisher Scientific). DNA was stained with DAPI for 1 min. Coverslips were added to glass slides using ProLong Gold mounting media (Molecular Probes, Thermo Fisher Scientific). The stained tissue was imaged with a Zeiss Axio Observer-Z1 widefield microscope equipped with a Zeiss Plan-Apochromat 63×/1.4 oil objective, an Axiocam 506 monochrome camera, and an Apotome setting. We acquired a *Z*-stack and used the Fiji software to make a maximum intensity projection for each image.

### Primary antibodies

We used the following antibodies: anti-ARL13B [immunofluorescence (IF), 1/500; WB, 1/1,000; NeuroMab, 11000053]; anti-HA (clone 3F10, IF:1/1000, 11867423001, Sigma-Aldrich); anti-NeuN (IF: 1:300, ab177487, Abcam); anti-DR6 (TNFRSF21; clone 6B6; IF, 1:200; MABC1594-25UL, Millipore); anti-ARL13B (IF, 1:500; Abcam, ab136648); anti-ARL13B (IF, 1:500; Proteintech, 17711-11AP); anti-Pericentrin (IF, 1:500; BD Biosciences, 611814); anti-Pericentrin (IF, 1:500; Abcam, ab4448); anti-NeuN (IF, 1:500; Abcam, ab177487); anti-GFP (IF, 1:1,000; Invitrogen, A11122); anti-V5 Tag (IF, 1:500; Invitrogen, R960-25); anti-adenylate cyclase 3 (IF, 1:500; Invitrogen, PA5-35382); anti-Ephrin-B1 (IF, 1:50; Bio-Techne, AF473); anti-Ephrin-B2 (IF, 1:50; Bio-Techne, AF496); anti-EphA4 (IF, 1:50; WB, 1:2,000; Proteintech, 21875-1-AP); anti-CSNK1G1 (IF, 1:50; OriGene, TA806333S); anti-SLC6A1 (IF, 1:500; Invitrogen, PA5-85766); anti-GABRG2 (IF, 1:100; Proteintech, 14104-1-AP); anti-DR6 (Tnfrsf21; IF, 1:100; Sigma-Aldrich, MABC1594); anti-SLC6A1 (IF, 1:500; Proteintech, 28488-1-AP); anti-GAPDH (WB, 1:20,000; Proteintech, 60004-1-Ig); anti-phospho-EPHA4-Tyr596 (WB, 1:2,000; St John's Laboratory, STJ90667); and anti-polyglutamylated tubulin (IF, 1:500; AdipoGen, GT335).

### Secondary antibodies

We used the following secondary antibodies, and their conditions of use include Alexa Fluor 488 goat anti-rabbit IgG (Invitrogen, A-11008, 1:500); Alexa Fluor 647 goat anti-mouse IgG1 (Invitrogen, A-21240, 1:500); Alexa Fluor 647 goat anti-mouse IgG2b (Invitrogen, A-21242, 1:500); Alexa Fluor 568 goat anti-mouse IgG2a (Invitrogen, A-21134, 1:500); Alexa Fluor 647 goat anti-rabbit IgG (Invitrogen, A27040, 1:500); Alexa Fluor 647 donkey anti-rabbit IgG (Invitrogen, A-31573, 1:500); Alexa Fluor 568 donkey anti-rabbit IgG (Invitrogen, A10042, 1:500); Alexa Fluor 647 donkey anti-mouse IgG (Invitrogen, A-31571, 1:500); Alexa Fluor 568 donkey anti-mouse IgG (Invitrogen, A10037, 1:500); Alexa Fluor 488 donkey anti-goat IgG (Invitrogen, A-11055, 1:500), Alexa Fluor 488 goat anti-mouse IgG2a (Invitrogen, A-21131, 1:500), Alexa Fluor 488 goat anti-rat IgG (Invitrogen, A-11006, 1:500); streptavidin, Alexa Fluor 488 (IF, 1:500; S11223, Invitrogen); and IRDye 680RD streptavidin (WB, 1:3,000; 926-68079, LI-COR Environmental).

### Lattice SIM

Primary neuronal cells were plated on high-precision type 1.5 coverslips. We incubated primary antibodies for 24 h at 4°C. We used Alexa Fluor secondary antibodies for each staining (IF, 1:500; Alexa Fluor 488, Alexa Fluor 568, Alexa Fluor 647; Molecular Probes, Thermo Fisher Scientific). These were incubated for 1 h 30 min at room temperature. We then mounted the coverslips on glass slides using ProLong Gold mounting media (Molecular Probes, Thermo Fisher Scientific). Our SIM acquisitions were done on a Zeiss Elyra 7 system equipped with lattice SIM technology. The signal was collected using a Zeiss Plan-Apochromat 63/1.4 oil objective. We kept the laser power and exposure time to a minimum to avoid photobleaching. In the ZEN imaging software, the SIM mode was used to process the acquired images. We used the line intensity scan function in the profile tab of the Zeiss Zen 2.3 (Blue Edition) software to generate intensity and colocalization graphs.

### Quantification and statistical analysis

Measurements of the cilia rate and candidate endogenous localization frequency or overexpression were performed on at least five randomly selected fields of cells per coverslip for each condition. At least two coverslips were analyzed per condition with similar confluence. The maximum intensity projections were used to perform this analysis with the Fiji software. Analysis was performed with the same signal parameters between candidates and their relative controls. Endogenous candidate localization frequencies measured on neuronal cilia were determined by the concurrent use of a neuronal marker or by morphological recognition of neurons and their cilia. Frequency measurements are an estimation. Localization of candidates was determined by the colocalization of fluorescent signals for the cilia channel and candidate channel. Data are reported as arithmetic means ± SEM, and statistical analysis was performed using paired *t* tests, nonparametric Mann–Whitney test and one-way ANOVA with *p* ≤ 0.05 as the cutoff for statistical significance. Statistical analyses were performed using the GraphPad Prism 9 software.

## Results

### Targeting biotin ligase to neuronal cilia in vivo

**Figure 1.**
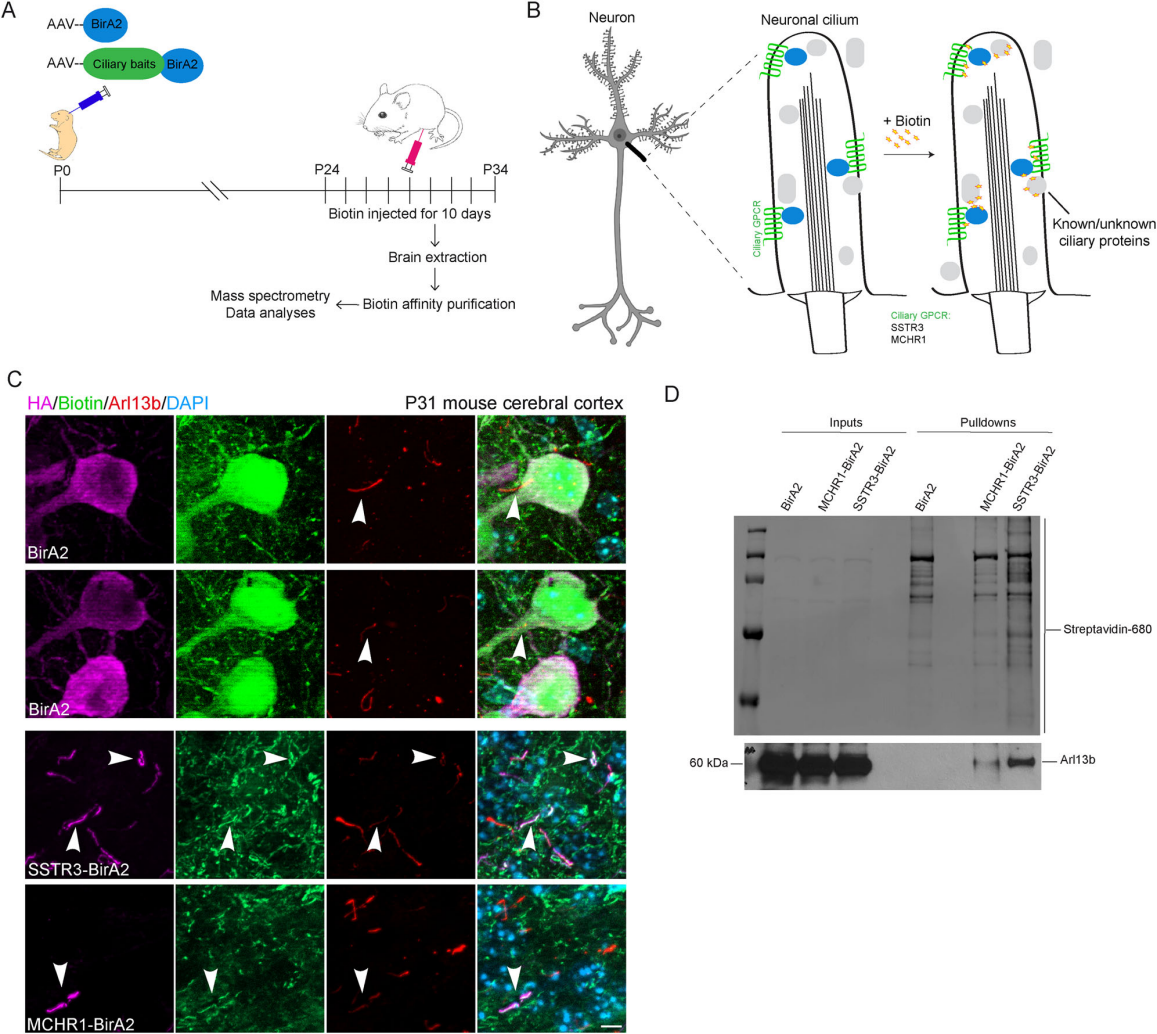
Targeting of the biotin ligase BirA2 to neuronal cilia in vivo. ***A***, Experimental procedure to label the protein composition of the neuronal cilia in the brain. ***B***, A simplified schematic of the targeting strategy for BirA2 using ciliary GPCRs. ***C***, Immunohistochemical labeling with antibodies to HA (fused to BirA2, magenta), biotin (green), and Arl13b (red) on mouse cerebral sections transduced with AAV-BirA2 or AAV-GPCRs-BirA2. DNA was stained with DAPI. Arrows point to neuronal primary cilia. Scale bars, 20 µm. ***D***, Streptavidin pulldowns were performed on total cell lysates from brains transduced with AAV-BirA2 or AAV-GPCRs-BirA2. WB were probed with Streptavidin-680 and Arl13b antibodies. See Extended Data Figures 1-1 and 1-2 for more details.

To understand how the neuronal cilium mediates neural functions, we focused on defining its intrinsic protein composition in the mouse brain (Fig. 1*A*,*B*). We deployed iBioID (Uezu et al., 2016), using a ciliary bait to target biotin ligase (BirA or BirA2; Roux et al., 2012; Kim et al., 2016) to neuronal cilia without affecting cilia biogenesis and length. We first tested several cilia-targeting baits: the N terminus of NPHP3 (a.a. 1–203; Mick et al., 2015), the ciliary targeting sequence of fibrocystin (Follit et al., 2010), and the ciliary GPCRs 5-HT6, MCHR1, and SSTR3; Hamon et al., 1999; Händel et al., 1999; Brailov et al., 2000; Schulz et al., 2000; Berbari et al., 2008a,b), to which we fused BirA or BirA2 (Roux et al., 2012; Kim et al., 2016). We initially expressed each of the baits in IMCD3 cells. In the presence of biotin, cells were serum starved for 48 h to induce cilia formation. We then assessed the subcellular localization of the baits and biotin labeling, as well as their effects on cilia assembly and length (Extended Data Fig. 1-1*A*). We next expressed the cilia-BioID constructs in the mouse brain, injecting purified AAVs coding for either HA-tagged BirA2 (or BirA) alone or each of the cilia-targeting BirA2 (−BirA) into lateral ventricles of neonatal mice at P0 (Fig. 1*A*; Extended Data Fig. 1-1), expressed under the *hSynapsin* promoter to drive the transgene's expression in neurons (Uezu et al., 2016). Injected mice recovered for at least 21 d and were then injected with 6.66 μM biotin daily for 10 d. At P31–P34, we extracted the mouse brains and generated sagittal sections of the mouse cerebral cortex. Sections were labeled with antibodies to biotin, ARL13B, and HA as a readout of the construct's expression. The most consistent and best performing baits in vitro and in vivo were the G-protein–coupled receptors (GPCRs) SSTR3 and MCHR1, which showed ciliary localization with positive biotin labeling (Fig. 1*C*; Extended Data Fig. 1-1). Note that BirA2 was fused to the C-terminus of SSTR3 and MCHR1 to localize within the intraciliary lumen. We therefore chose to utilize these GPCRs as baits, with a focus on mapping the molecular networks within the neuronal cilium. Compared with the MCHR1 bait, SSTR3 had better coverage of the number of cilia labeled, consistent with an overall higher biotinylation profile (Fig. 1*C*,*D*). It is important to note that the same AAV backbone has been previously validated as expressing the BirA2 fusions exclusively in neurons, under the control of the hSynapsin promoter (Uezu et al., 2016). We also assessed the extent of and specificity of biotin labeling throughout the whole brain for BirA2 alone and GPCRs-BirA2. We found that fusion protein expression consistently colocalizes with biotin labeling. The most prominent sections expressing these constructs were from the cerebral cortex (Fig. 1*C*) and, to a lesser extent, the hippocampus, with limited expression in the cerebellum. We therefore kept the anterior mouse brain and removed the cerebellum for these studies.

10.1523/JNEUROSCI.0800-24.2025.f1-1 Figure 1-1 Testing of ciliary baits to target biotin ligaseA. A summary of tested ciliary baits to target the biotin ligase to neuronal cilia in vivo. Baits were first tested in IMCD3 cells and then assessed in the mouse brain, as detailed in figure 1A. Immunohistochemical labeling from mouse brain sections transduced withB. AAV-NPHP3 (a.a 1-203)-BirA.C. AAV- ciliary targeting sequence of Fibrocystin (CTS)-BirA2.D. AAV-5HTR6-BirA2. Sections are labeled with antibodies to Biotin (green), ciliary markers ACIII and Arl13b (red), and HA (magenta). DNA was stained with DAPI. Scale bars: 20  µm. Download Figure 1-1, TIF file.

**Figure 2.**
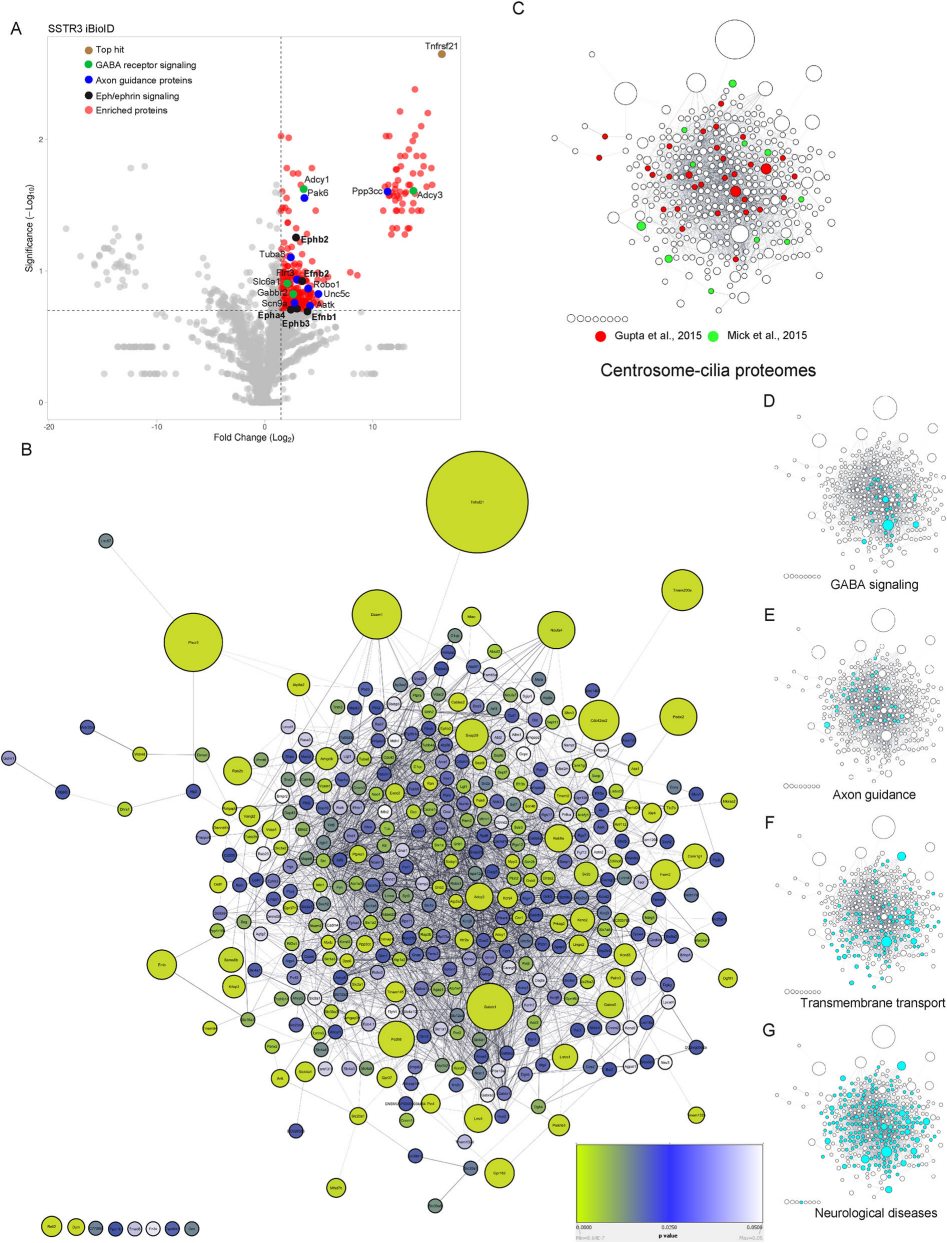
Cilia iBioID identified neural-specific clusters in neuronal cilia in vivo. ***A***, A volcano plot of SSTR3-proximate proteins labeled by BioID. The red dots show the proteins considered hits, delimited by the selected thresholds: log2 fold change ≥ 2 and significance ≥ 0.7. The top hit, Tnfrsf21, is highlighted in brown; GABA receptor signaling in green, axon guidance proteins in blue, and Eph/Ephrin signaling in black. The *X*-axis denotes the log2 fold change of SSTR3-BirA2: BirA2 control. In the *Y*-axis, significance displays the negative log10 transformed *p* value for each protein. ***B***, The neuronal ciliary network. This is a clustergram topology of all proteins enriched in SSTR3 iBioID. It is composed of protein networks, with proteins shown as spheres and their interactions as lines. The Cytoscape software was deployed to generate this network, relying on its mining capabilities to extensively search the literature. The size of each sphere is correlated to the fold change value and the color shade to the *p* value. ***C***, Clustergram topology showing overlap of SSTR3 iBioID hits with other published centrosome–cilia proteomes [Gupta et al. (2015), red, and Mick et al. (2015), green]. ***D–G***, Clustergram topologies of hits associated with the indicated functional category (cyan). See Extended Data Figures 2-1–2-7 for more details.

10.1523/JNEUROSCI.0800-24.2025.f1-2 Figure 1-2 Native biotin labeling in BirA2 and GPCRs-BirA2 brainsA. ­Immunohistochemistry of P31 cortical slices transduced with AAV-BirA2 and labeled with antibodies to HA (fused to BirA2, magenta), Biotin (green), and Arl13b (red). DNA was stained with DAPI. Scale bars: 10  µm. Images were adjusted to approximately match the intensity range of the GPCR-BirA2 images (Figure 1C). Panels show high BirA2 expression and biotin labeling.B. same as A, with low BirA2 expression and biotin staining in the midbrain area.C. ­Immunohistochemistry of P8 brain sections transduced with AAV-BirA2 or GPCRs-BirA2 and labeled with antibodies to HA (fused to BirA2, magenta), biotin (green), and Arl13b (red). DNA was stained with DAPI. Scale bar: 20  µm. Images show high BirA2 expression with low biotinylation. Download Figure 1-2, TIF file.

We note that the GPCR-BirA2 brains exhibited higher apparent background biotinylation than that visible in control samples. As in prior iBioID studies (Uezu et al., 2016), we use soluble BirA as a control condition, which results in broad biotinylation throughout the entire neuron. The widespread nature of this signal can obscure the visibility of background biotinylation in these samples. Therefore, we adjusted the intensity range of the BirA2 image to approximately match the other two conditions, revealing a similar biotin background (Extended Data Fig. 1-2*A*). Additionally, we examined the midbrain region, where overall BirA2 expression and biotin labeling are lower in all samples due to reduced AAV transduction further from the lateral ventricles. In this region, we observed similar background biotinylation across all samples, regardless of the bait used or the age of the animal (Extended Data Figs. 1-1*B*–*D*, 1-2*B*). To further validate these findings, we injected P1 pups with AAVs encoding BirA2 alone and GPCRs-BirA2. After 48 h, biotin was administered four times over 4 d, which was insufficient to induce a prominent biotinylation pattern. Brains were perfused and collected at stage P8, and sections were stained with streptavidin and antibodies against HA and Arl13b. Under these conditions, the fusions were correctly expressed and targeted, but due to the reduced levels of biotin administered, this exogenous labeling was greatly reduced. In all conditions, we consistently found the same native biotin background. This further reinforces our conclusion that BirA2 controls exhibit comparable levels of similarly localized background biotinylation to GPCR-BirA2 conditions, but this is often visually obscured by the broad nature of the induced biotinylation in the control compared with the highly cilia-targeted GPCR-BirA2 conditions (Extended Data Fig. 1-2*C*).

iBioID has been previously applied to the synaptic compartment of the brain (Uezu et al., 2016). To better capture ciliary baits, we partially modified the original iBioID experimental procedure (detailed in Materials and Methods). Three key changes were made to the prior iBioID: (1) a greater number of AAV particles were injected; (2) dual-site introduction of biotin solution was combined with longer incubations; and (3) a less harsh lysis buffer was used during the purification stage. These changes have been key to successfully enriching biotinylated proteins from MCHR1- and SSTR3-BirA2 pulldowns. GPCR-BirA2 pulldowns show a clear enrichment of the ciliary marker ARL13B not observed in the BirA2 alone condition (Fig. 1*D*). Thus, we have found that GPCRs are the most effective baits for our subsequent experiments. Our experimental approach addresses the challenge of identifying low-abundance ciliary proteins and can be used for low-abundance neural proteomes or cilia subtypes in the anterior mouse brain.

### Neuronal cilia BioID2 unveils novel ciliary nodes with a subset of known ciliary proteins

To obtain a robust set of hits, quantitative LC–MS/MS was performed for each sample (BirA2, SSTR3-BirA2, and MCHR1-BirA2) in biological triplicates (seven mice per biological replicate). We considered as hits all proteins with at least a twofold enrichment (with a *p* value of <0.05) in GPCRs-BirA2 compared with BirA2 alone. A total of 389 proteins were enriched in the SSTR3-BirA2 dataset compared with BirA2 alone (Fig. 2*A*; Extended Data Fig. 2-3). Only 12 hits were enriched in the MCHR1 dataset (Extended Data Figs. 2-1*A*, 2-3), consistent with insufficiently robust MCHR1 labeling (Fig. 1*D*). Additionally, we identified 8 out of 12 hits from the MCHR1 iBioID that were found in the SSTR3 iBioID. Hence, we focused our analyses on the SSTR3 iBioID dataset (cilia iBioID dataset).

10.1523/JNEUROSCI.0800-24.2025.f2-1 Figure 2-1 Volcano plots of SSTR3 and MCHR1 BioID and GO analysis**A.** Volcano plot of MCHR1-proximate proteins labeled by BioID. The red dots show the proteins considered hits, delimited by the selected thresholds: log2 fold change ≥ 2 and significance ≥ 0.7. The X-axis denotes the log2 fold change of MCHR1-BirA2: BirA2 control. In the Y-axis, significance displays the negative log10 transformed p-value for each protein.**B.** Volcano plot of SSTR3-proximate proteins labeled by BioID as shown in figure 2A. The green dots highlight known ciliary proteins overlapping with cilia proteomes discovered in Gupta et al., 2015 and Mick et al., 2015.**C.** Gene ontology analysis of hits enriched in SSTR3 iBioID. The table summarizes the most enriched signaling pathways with their corresponding p-values. Download Figure 2-1, TIF file.

10.1523/JNEUROSCI.0800-24.2025.f2-2 Figure 2-2 Insights into connections between neuronal cilia and brain disorders**A.** Clustergram topology of hits associated with cognitive impairment (orange), autism (green), and neurodevelopmental disorders (NDD, red)**B.** Clustergram topology of proteins associated with seizure (orange), ataxia (green), and other movement disorders (red)**C.** Clustergram topology of hits associated with Parkinson’s disease (orange), Huntington’s disease (green), and Alzheimer’s disease (red). For all clustergrams, overlapping hits were shown in one of the categories. Download Figure 2-2, TIF file.

**Figure 3.**
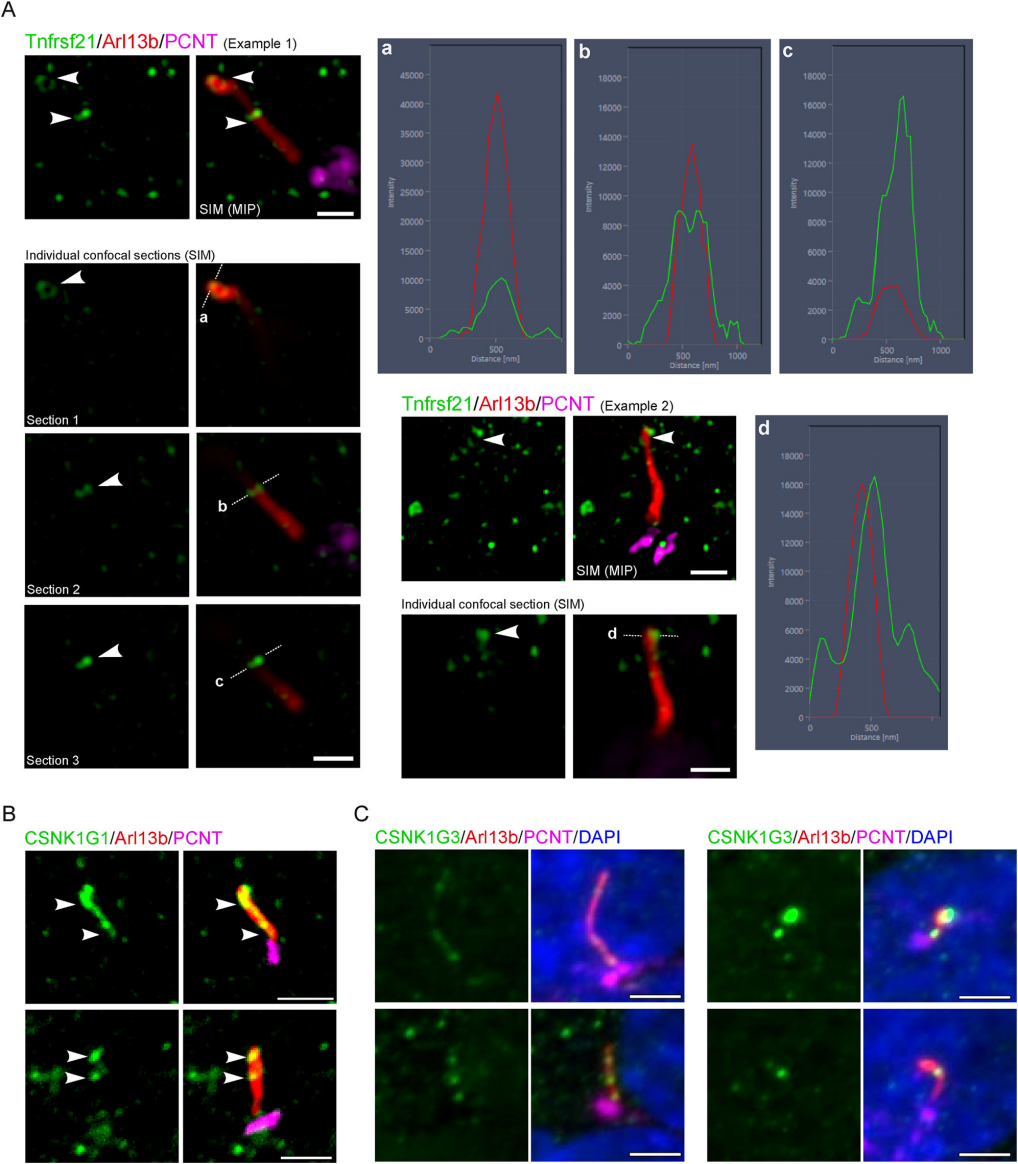
Selected iBioID hits localize to neuronal primary cilia by immunofluoresence. ***A***, SIM acquisitions show that the top hit, Tnfrsf21, localizes to the ciliary tip of primary mouse cortical neurons. Two representative examples are shown with cilia by Arl13b (red), centrosomes using Pericentrin (magenta), and Tnfrsf21 (green). Individual confocal sections were analyzed with the line intensity scan function, illustrated with dashed lines, to generate fluorescence spectra graphs [Intensity (A.U.) by distance (nm)], showing the intensity profiles of Tnfrsf21 (green) and Arl13b (red). Scale bars, 500 nm. ***B***, CSNK1G1 and (***C***) CSNK1G3 are labeled in primary cortical neurons and IMCD3 cells, respectively, with Arl13b (red) and Pericentrin (magenta). DNA was stained with DAPI (blue). Scale bars, 1 µm.

10.1523/JNEUROSCI.0800-24.2025.f2-3 Figure 2-3 Quantitative proteomics results of control and GPCRs iBioID. Download Figure 2-3, XLSX file.

**Figure 4.**
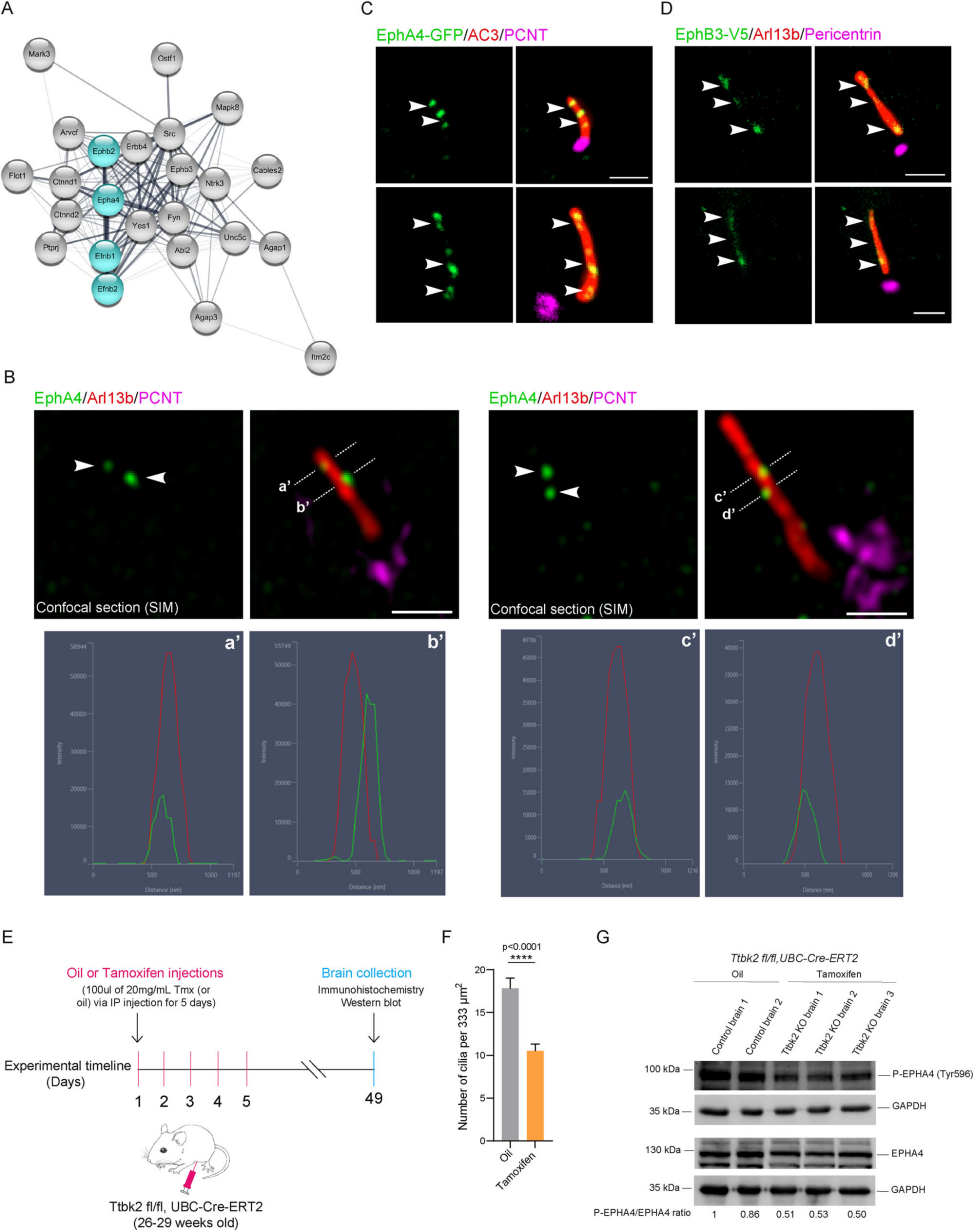
Primary cilia harbor Eph/Ephrin signaling. ***A***, Clustergram topology of Eph/Ephrin proteins enriched in the cilia iBioID dataset. Most of the highlighted hits are validated in the following sections of this figure. ***B***, Representative immunofluorescence confocal images acquired with the structured illumination microscopy (SIM). Wild-type primary cortical neurons are labeled with EphA4 (green) localizing to cilia costained with the ciliary marker Arl13b (red) and centrosomal marker Pericentrin (magenta). Arrows indicate areas of colocalization of EphA4 and Arl13b or Pericentrin. Dashed lines indicate the selections used to generate fluorescence spectra graphs [***a***, ***b***, ***c***, and ***d***; graphs showing intensity (A.U.) by distance (nm)], illustrating the intensity profiles of EphA4 (green) and Arl13b (red). Scale bars, 1 µm. ***C***, Representative immunofluorescence images of overexpressed EphA4-GFP (green) in IMCD3 cells. Primary cilia are labeled with the ciliary marker ADCY3 (red) and centrosomal marker Pericentrin (magenta). Arrows show ciliary localization of EphA4-GFP. Scale bars, 1 µm. ***D***, Representative immunofluorescence images of overexpressed EphB3-V5 (green) in IMCD3 cells. Primary cilia are labeled with the ciliary marker ADCY3 or Arl13b (red) and centrosomal marker Pericentrin (magenta). Arrows show ciliary localization of EphB3-V5. Scale bars, 1 µm. ***E***, Experimental procedure to genetically ablate cilia in mature brains. ***F***, The graph shows the mean number of primary cilia in the motor cortex from oil or tamoxifen-injected Ttbk2^fl/fl^;Ubc-Cre-ERT2+ male mice. A statistical comparison was performed using the nonparametric Mann–Whitney test. The *p* value is shown on the graph. Fields of view of 333 µm^2^ from the motor cortex were selected between oil (12 fields of view) and tamoxifen-injected (21 fields of view) mice. ***G***, WB from total brain lysates from oil or tamoxifen-injected Ttbk2^fl/fl^;Ubc-Cre-ERT2+ male mice. Each of the brains comes from a separate animal. Blots are probed with antibodies to Phospho (P)-EphA4 (Tyr596) and EPHA4. GAPDH was used as a loading control. Bands were quantified with the area under the curve using the Fiji software. See Extended Data Figure 4-1 for more details.

10.1523/JNEUROSCI.0800-24.2025.f2-4 Figure 2-4 Gene ontology analysis of SSTR3-iBioID hits showing categories of protein functions and their corresponding p-values within the dataset. Download Figure 2-4, XLSX file.

**Figure 5.**
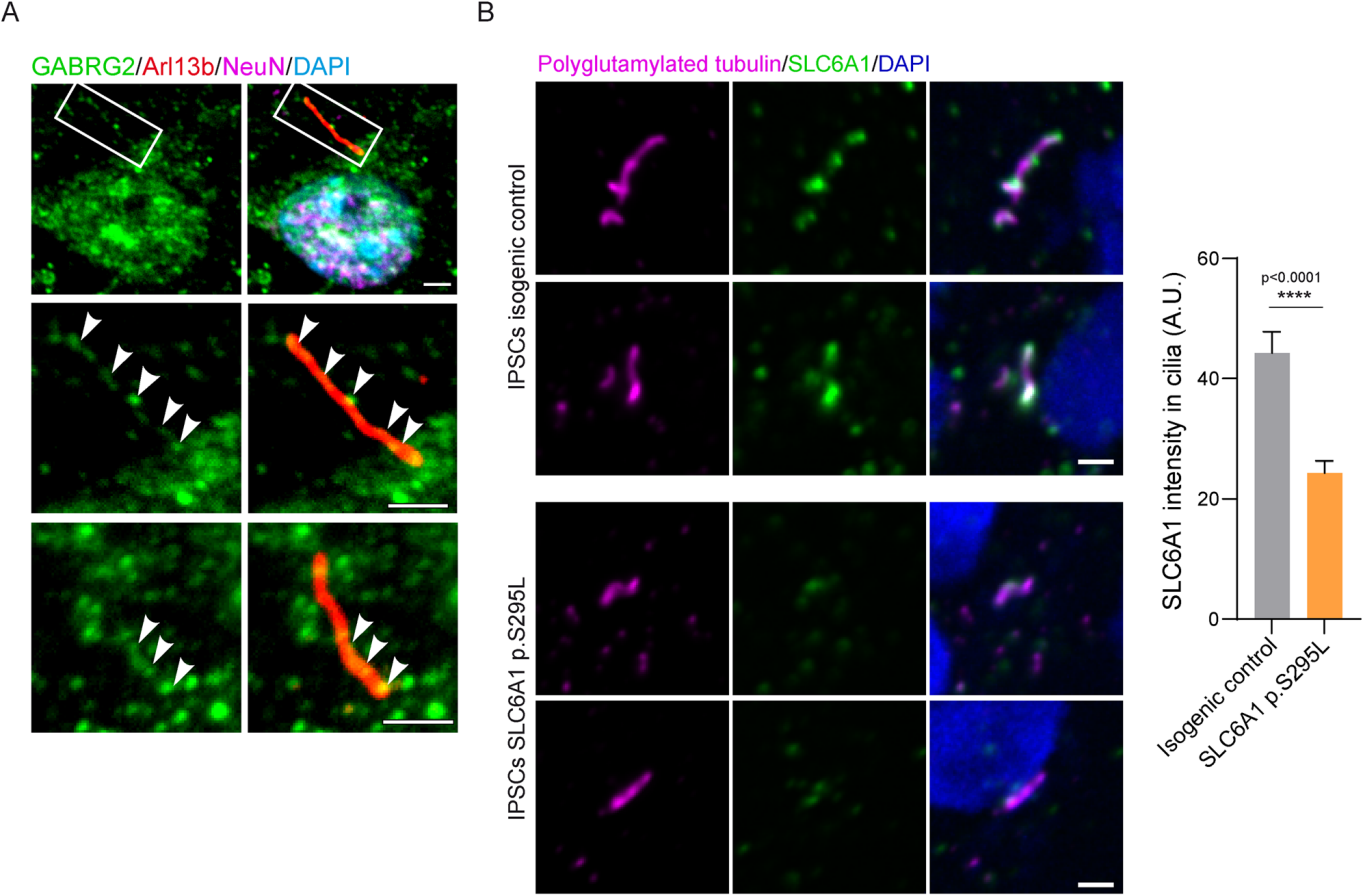
Effectors of GABAergic signaling reside within neuronal primary cilia. ***A***, Representative immunofluorescence images of GABRG2 (green) localizing to neuronal primary cilia labeled with Arl13b (red). Wild-type neurons are labeled with and identified by NeuN (magenta). Arrows indicate areas of colocalization of GABRG2 and Arl13b. Scale bars, 1 µm. ***B***, Representative immunofluorescence images of SLC6A1 (green) in IPSC line derived from a patient carrying the SLC6A1 p.S295L mutation with its counterpart isogenic control. Primary cilia and centrosome are labeled with polyglutamylated in magenta. DNA was stained with DAPI (blue). Scale bars, 2 µm. The graph shows the mean SLC6A1 intensity in cilia. Statistical analysis was performed using the nonparametric Mann–Whitney test. The *p* value is shown on the graph. Quantification was done on 91 cells for the IPSC isogenic control and 88 cells for the IPSCs SLC6A1 p.S295L.

10.1523/JNEUROSCI.0800-24.2025.f2-5 Figure 2-5 Analysis of Diseases and functions categories of hits enriched in SSTR3-iBioID dataset. Download Figure 2-5, XLS file.

10.1523/JNEUROSCI.0800-24.2025.f2-6 Figure 2-6 Most enriched canonical pathways that overlap with our SSTR3-iBioID. Download Figure 2-6, XLS file.

10.1523/JNEUROSCI.0800-24.2025.f2-7 Figure 2-7 An exhaustive list of disease categories and their associated genes, enriched in the SSTR3-iBioID dataset. Download Figure 2-7, XLS file.

To identify and prioritize signaling networks enriched in neuronal cilia, we performed GO analysis. We first used Gonet (Pomaznoy et al., 2018) to determine categories of molecular functions of all cilia hits through GO term enrichment analysis. Each of the categories was generated and linked to the representative genes with a *q* value threshold of ≤0.05 (Extended Data Fig. 2-4). Most of the proteins identified are transmembrane transporters, ion channels, and receptors, consistent with SSTR3-BirA2 labeling capturing components in proximity to the ciliary membrane. Using the IPA database, we found significant enrichment of pathways implicated in cell-to-cell interaction and cellular morphology (Extended Data Fig. 2-5). We also employed Cytoscape to reconstruct the molecular interactome of the cilia dataset. The neuronal ciliary network is constituted by interconnected spheres, in which the size of each sphere is correlated to the fold change value and the color shade to the *p* value (Fig. 2*B*).

We then compared our hits with known centrosome–cilia proteomes obtained in vitro in mammalian immortalized cells (Gupta et al., 2015; Mick et al., 2015). We uncovered a total of 42 proteins (10.8%), overlapping with our cilia iBioID dataset (Fig. 2*C*; Extended Data Figs. 2-1*B*, 2-3). These results also suggest overlapping protein repertoires between neuronal cilia and those of immortalized cells. While we used SSTR3, a membrane-associated protein, as bait, we still detected ciliary proteins from other ciliary compartments. This includes early ciliary vesicles as well as the microtubule-based axoneme. Early cilia initiation regulators such as SNAP29, EHD1, EHD3, and RAB8A were enriched in the cilia dataset (Nachury et al., 2007; Lu et al., 2015). Potential hits related to the axonemal compartment have been identified, such as the subunit of kinesin II, KIFAP3, and specific tubulin subunits, including TUBB2A, TUBB2B, and TUBB4B (Nakayama and Katoh, 2018; Mechaussier et al., 2022; Mollica et al., 2023; Extended Data Fig. 2-3). Modulators of the cilium's length and stability, such as SEPT6, SEPT7, and SEPT11, were found in our cilia hits (Kanamaru et al., 2022). GPCR downstream effectors such as ADCY3 and ADCY9 were also detected (Extended Data Figs. 2-1*B*, 2-3). Note that ADCY3 is a well established marker for neuronal cilia in the brain (Bishop et al., 2007; Berbari et al., 2008a,b; Guadiana et al., 2016). We also identified the protein kinase cAMP-activated catalytic subunit beta, PRKACB, which is one of the subunits of the holoenzyme PKA required for SHH signaling (Tuson et al., 2011). Similarly, the noncanonical Wnt target genes PLCB1 and DAAM1 were hits in our cilia dataset (Saito et al., 2015; Extended Data Fig. 2-3). Overall, our findings from the SSTR3 iBioID covered compartments and signaling pathways previously linked to primary cilia function. This indicates that our updated iBioID experimental procedure successfully captured known ciliary proteins in vivo from the brain.

More importantly, 89.2% of the identified hits were not previously linked to primary cilia. We therefore concentrated on identifying signaling pathways that could provide insights into novel ciliary nodes in the brain. We uncovered the gamma-aminobutyric acid (GABA) receptor signaling as the top pathway, including 27 components enriched in our neuronal cilia dataset (Fig. 2*D*; Extended Data Figs. 2-1*C*, 2-6). GABA is a major inhibitory neurotransmitter in the mammalian brain (Wu and Sun, 2015). Our cilia dataset contained eight subspecific GABA receptors that include the GABA_A_ receptors, GABRG2 and GABRA1, and the GABA_B_ receptor, GABBR1. We also found 39 proteins involved in axon guidance signaling (Fig. 2*E*; Extended Data Figs. 2-1*C*, 2-6). This is consistent with previous studies showing that neuronal ciliary signaling is critical for axonal guidance and branching complexity (Kumamoto et al., 2012; Guo et al., 2017, 2019; Bowie and Goetz, 2020). It is worth noting that the neuronal ciliary network contained 92 proteins implicated in transmembrane transport (Fig. 2*F*; Extended Data Fig. 2-4), which could be involved in key neuronal processes, such as neurotransmitter release and reuptake and synaptic plasticity.

### Cilia iBioID underscores ciliary links to neurological disorders

To uncover new putative linkages between neuronal cilia and human neurological disorders, we again employed the IPA database (Extended Data Fig. 2-3). We found that at least 202 of the cilia-enriched hits are mutated in or associated specifically with neurological disorders (IPA; *p* value range, 6.65 × 10^−6^–4.03 × 10^−28^; Fig. 2*G*; Extended Data Figs. 2-2, 2-7). In contrast, the background hits depleted in SSTR3 iBioID (or enriched in BioID2 dataset) were not associated with brain disorders but rather cancer (120 molecules; IPA; *p* value range, 1.8 × 10^−2^–2.37 × 10^−28^). We provide a comprehensive analysis of the cilia dataset, shown as categories of diseases and functions accompanied by their *p* values (Extended Data Fig. 2-6). From these categories, we chose key diseases/functions and overlapped their associated proteins into our neuronal ciliary network to generate clustergrams (Fig. 2; Extended Data Fig. 2-2). About 95 genes were linked to movement disorders and motor dysfunction (Extended Data Figs. 2-2*A*, 2-7), which was previously observed in several ciliopathies and cilia-related spinocerebellar ataxia (Reiter and Leroux, 2017; Bowie et al., 2018; Bowie and Goetz, 2020). A recent study demonstrated that neuronal primary cilia are disrupted in the dentate gyrus of fragile X syndrome, which is the most common monogenic cause of autism spectrum disorder (Lee et al., 2020). We therefore overlapped our dataset with the SFARI Autism database (https://gene.sfari.org/database) of genes directly linked to the autism spectrum disorder (Extended Data Fig. 2-2*B*). We found 64 genes present in our neuronal cilia dataset, indicating a deeper yet underexplored linkage between autism and primary cilia function in the brain. Similarly, we uncovered 43 genes that were related to disorders with cognitive impairment (Extended Data Fig. 2-2*B*), considered one of the cardinal features of ciliopathies (Valente et al., 2014). Increasing evidence highlights defects in primary cilia structure and function in neurodegenerative disorders. These connections have been reported particularly for Alzheimer's, Huntington's, and Parkinson's disorders (Keryer et al., 2011; Dhekne et al., 2018; Nguyen et al., 2021; Sobu et al., 2021; Ma et al., 2022); however, the cellular and molecular mechanisms underlying these links have yet to be uncovered. Our findings revealed for the first time that many molecules associated with these neurodegenerative disorders likely reside in the ciliary membrane of neurons (Extended Data Fig. 2-2*C*). Altogether, our results constitute a foundation of new information about the molecular composition of neuronal cilia with novel connections to brain disorders.

### Validation of iBioID hits in mouse primary neurons

We began validating these findings by examining the subcellular localization of the top hit of cilia iBioID (Fig. 2*A*). Tnfrsf21, or death receptor 6 (DR6), is a member of the tumor necrosis factor receptor superfamily and known to mediate axonal plasticity (Pan et al., 1998; Marik et al., 2013; Gamage et al., 2017). We used primary antibodies against endogenous Tnfrsf21 in mouse primary cortical neurons, costaining with ARL13B and Pericentrin to label primary cilia and the centrosome, respectively. Using super-resolution microscopy, we have observed Tnfrsf21 in an atypical ring-shaped pattern at the distal cilium (63.60% ± 5.97 of cilia positive for Tnfrsf21). We analyzed individual confocal sections and generated line intensity scan graphs, confirming colocalization between Tnfrsf21 and Arl13b (Fig. 3*A*). Such discrete subcellular localization would have been difficult to uncover without our iBioID findings. Additionally, we have found that two out of three members of a Casein Kinase 1 (CK1) subfamily, CK1 gamma (CK1G), CSNK1G1 and CSNK1G3, were significantly enriched in our dataset, and upon examining their subcellular localization by immunofluorescence, we found both CSNK1G1 and CSNK1G3 to be highly specific to neuronal primary cilia (Fig. 3*B*,*C*). CK1 is known to modulate fast synaptic transmission, mediated by glutamate, the brain excitatory neurotransmitter (Chergui et al., 2005). Our data are also consistent with the previously described ciliary localization of CSNK1G1 (Li et al., 2016). Overall, the tested hits showed discrete localization throughout the neuronal cilium. This confirms that iBioID successfully identified novel cilia-resident receptors as a promising first step to defining ciliary signaling in the brain.

### Eph/Ephrin signaling components localize to neuronal primary cilia

As part of the iBioID axonal guidance module, we isolated the Ephrin/Eph cluster including ephrin ligands and receptors (Fig. 4*A*). We assessed the subcellular localization of three proteins, including two ephrin ligands, EFNB1 and EFNB2, as well as the Eph receptor, EPHA4, by immunofluorescence labeling in primary cortical neurons. We costained the protein of interest with ARL13B and Pericentrin. All three tested proteins exhibit a similar and intriguing ciliary localization of a two-foci pattern along the neuronal cilium (the percentage of positive cilia for Efnb1, 72.96% ± 5.97; Efnb2, 85.36% ± 1.69; EphA4, 92.57% ± 4.28). On confocal (or apotome) images, we performed line scan analysis and found that all three proteins colocalize or partially colocalize with Arl13b (Fig. 4*B*; Extended Data Fig. 4-1). To further confirm the ciliary localization of EphA4, we transiently expressed a GFP fusion in IMCD3 cells. We next performed immunofluorescence with antibodies to GFP, the ciliary marker ARL13B, and centrosomal protein Pericentrin. Consistent with the endogenous labeling, EphA4-GFP was observed in distinct foci throughout the cilium. However, we detected more than two foci, which may be due to overexpression or a different regulation in the non-neuronal IMCD3 cells (Fig. 4*C*). In contrast, EphB3-V5 showed a more uniform ciliary localization in IMCD3 cilia (Fig. 4*D*).

10.1523/JNEUROSCI.0800-24.2025.f4-1 Figure 4-1 Efnb1 and Efnb2 are present in neuronal cilia Representative immunofluorescence confocal or apotome images of **A.** Efnb1 and **B.** Efnb2 (green) in neuronal primary cilia labeled with the ciliary marker Arl13b (red) and centrosomal Pericentrin (magenta). Arrows indicate the colocalization of Ephrins with Arl13b. Dashed lines indicate the selections used to generate fluorescence spectra graphs (Graphs showing intensity (A.U.) by distance (µm)), illustrating the intensity profiles of Efnb1 or Efnb2 (green) vs. Arl13b (red). Scale bars: 1  µm. Download Figure 4-1, TIF file.

We then investigated the role of primary cilia in mediating Eph/Ephrin signaling in the adult brain. To this end, we conditionally ablated cilia by knocking out a key cilia assembly kinase, TTBK2, in the mature brain (Bowie and Goetz, 2020) to assess the status of EPHA4 activation. Since our iBioID was performed in mature brains, we utilized *Ttbk2^fl/fl^*,*UBC-Cre-ERT2* adult mice (26–29 weeks of age), which received either oil or tamoxifen through intraperitoneal injections. Brains were collected for analysis 6 weeks postinjections (Fig. 4*E*). We first quantified cilia in the motor cortex, comparing oil and tamoxifen-injected brain sections. We found that *Ttbk2 KO* brains showed about a 50% cilia loss (Fig. 4*F*). We hence measured by WB the levels of phospho-EPHA4 (Tyr596) that we normalized to the total levels of EPHA4. Our analyses showed a reduction in the P-EPHA4/total EPHA4 ratio in all *Ttbk2 KO* brains compared with control brains (Fig. 4*G*). This suggests that primary cilia mediate the activation of EPHA4 signaling in the mature brain.

### Neuronal cilia harbor key regulators of GABAergic neurotransmission

GABA signaling was our top signaling pathway enriched in the neuronal ciliary network (Fig. 2*D*; Extended Data Fig. 2-1*C*). We therefore evaluated the subcellular localization of one of the GABA_A_ receptors, GABRG2, implicated in GABAergic neurotransmission of the mammalian central nervous system. By immunofluorescence, GABRG2 showed a consistent, punctate signal throughout the cilium in mouse primary cortical neurons (GABRG2, 77.92% ± 3.52; Fig. 5*A*). We also assessed a key GABA transporter, SLC6A1, that mediates GABA reuptake in neurons. Mutations in the Slc6a1 gene are associated with neurodevelopmental disorders characterized by intellectual disability, epilepsy, and autism spectrum disorder (Goodspeed et al., 2020). For example, the SLC6A1 p.S295L mutation is considered a loss function, causing disrupted protein folding and intracellular retention and degradation of the variant. As a result, there is less functional SLC6A1 transporter at the membrane, which significantly decreases GABA intake (Mermer et al., 2021; Lindquist et al., 2023). To assess SLC6A1 subcellular localization, we utilized an IPSC line derived from a patient carrying the SLC6A1 p.S295L mutation analyzed with its counterpart isogenic control (Nwosu et al., 2022). We found that SLC6A1 localized to cilia in control cells (Fig. 5*B*, top panel). In the presence of the mutation, we observed reduced levels of ciliary SLC6A1, indicating that the loss-of-function mutation may have affected the trafficking of the variant to the cilium (Fig. 5*B*, bottom panel). This also validates the specificity of the SLC6A1 antibody.

## Discussion

In this work, we report the successful adaptation of proximity labeling to capture the protein constituents of primary cilia in an in vivo context for the first time. iBioID is a critical first step to understanding the molecular mechanisms by which neuronal primary cilia mediate neural function and how their perturbations can lead to neurological disorders. Neuronal cilia have been recently linked to serotoninergic signaling and reported to form a novel type of synapse called the axo-ciliary synapse (Sheu et al., 2022). While our cilia iBioID dataset does not have cell-type–level resolution to distinguish between neuronal subtypes associated with specific neurotransmitters such as serotonin, dopamine, and glutamine, we identified several hits related to synaptic structure and synaptogenesis. These findings are consistent with previous reports linking neuronal cilia to the regulation of synaptic connectivity (Kumamoto et al., 2012; Guo et al., 2017; Bowie and Goetz, 2020; Tereshko et al., 2021; Sheu et al., 2022). For example, loss or dysfunction of neuronal cilia (*Ttbk2* or *Ift88* conditional mutants) causes loss of excitatory synapses on Purkinje neurons from the climbing fibers in the adult cerebellum (Bowie and Goetz, 2020). Therefore, our data combined with previous evidence highlight a potential molecular cross talk between ciliary networks and neuronal synapses.

Many canonically studied ciliary receptors are very highly concentrated within cilia and are present only at very low levels elsewhere in the cell (Corbit et al., 2005; Loukil et al., 2021; Sheu et al., 2022). One significant aspect of our approach is our identification of cilia-residing signaling molecules that are present within the ciliary membrane at lower levels and those that are found in cilia as well as other cellular locations, including the plasma membrane. This is the case for the EphR/Ephrin signaling molecules identified through this study, as well as GABA receptors. This is also true for other receptors recently identified within cilia, including the mu opioid receptor (Fagan et al., 2024) and GPR158 (Rivagorda et al., 2025), a GPCR that we also identified in our dataset. Such dual ciliary and plasma membrane-residing receptors have not been extensively studied previously. However, these receptors represent an important category of ciliary proteins, implying dynamic trafficking between the cilium and other cellular compartments. Examining how such dynamic trafficking is regulated as well as how receptor activation at the cilium might activate specific downstream signaling distinct from activation at the plasma membrane will be important questions to address in our future work.

One of our key results identified several Eph/Ephrin signaling proteins as residing in neuronal cilia. The Eph/Ephrin signaling mediates several critical intercellular interactions during embryonic development and adulthood. It modulates cytoskeleton organization, cell morphology, adhesion, migration, and survival (Pasquale, 2008; Coulthard et al., 2012; Klein, 2012; Verma et al., 2023). More importantly, Eph/Ephrin interactions occur on the cell surface, which can drive forward and reverse signaling at synapses and other subcellular localizations. While Eph/Ephrin can act as cues for attractive and repulsive axonal guidance, cilia have also been shown to regulate axonal pathfinding (Guo et al., 2019; Dumoulin et al., 2024). There is therefore a possibility of molecular cross talk between cilia and synapses. Our data point to possible regulation of Eph receptors by cilia in the brain. We observed a reduction in EphA4 phosphorylation/activation upon cilia ablation in *Ttbk2* KO brains. TTBK2 is a well known regulator of cilia assembly and the SHH pathway, acting upstream of IFT proteins (Goetz et al., 2012; Čajánek and Nigg, 2014; Lo et al., 2019). Additionally, the *Ttbk2* conditional knock-out has been shown to phenocopy the *Ift88* conditional knock-out in the mature mouse cerebellum, highlighting their overlapping abilities to disrupt cilia structure and signaling in neural tissues (Bowie and Goetz, 2020). EphA4's downstream signaling cascades are known to modulate synaptic plasticity, axon guidance, and neuronal circuit formation (Pasquale, 2008). In this context, the localization of EphA4 to the ciliary signaling hub might allow precise spatial control leading to finely tuned activation by Ephrin ligands, which in turn changes cell morphology, migration, and adhesion. Further studies will be necessary to comprehensively identify the EPHA4 downstream signaling mediated by cilia. It is plausible that cilia-localized Eph/Ephrin signaling components may be among the molecular effectors acting as focal points for cellular interaction, promoting privileged cell–cell communication and signaling. We speculate that Eph/Ephrin-expressing cilia may drive fine morphological changes that affect the intercellular communication within the mature brain.

We found the GABA inhibitory pathway to be our top-enriched pathway. Consistent with our data, a GABA_B_ receptor was found at the ciliary base in mouse pancreatic islets of Langerhans. Upon agonist binding, the receptor translocated into cilia, driving selective Ca^2+^ influx (Sanchez et al., 2022). Our cilia iBioID GABA receptors may similarly regulate calcium influx in neuronal cilia, which can affect synaptic transmission and plasticity (Catterall and Few, 2008). Recent studies showed that acute disruption of ARL13B enhances the excitatory capacity of pyramidal neurons (Tereshko et al., 2021). This may indicate an inhibitory role for primary cilia, which is consistent with our data. It is likely that specific focal points on neuronal cilia either release or receive GABAergic signaling, which in turn may lead to intracellular changes within the receiving neuron. In contrast with Eph/Ephrin subcellular localization, SLC6A1 localization was more uniform across the primary cilium. A human disease-associated mutation in SLC6A1 (p.S295L; Mermer et al., 2021; Nwosu et al., 2022) disrupted the ciliary localization of this GABAergic transporter in IPSC patients’ cells. Our findings unveil previously unknown neural ciliary hits that can be relevant in a neuropathological context. The connection between SLC6A1 and cilia will have to be further explored in relevant cell types, including neural stem cells and differentiated neurons.

Recent studies have reported that neuronal cilia can play a role in cell–cell communication between neurons and astrocytes. The absence of cilia in neurons in the brain led to significant changes in astrocytes, which became reactive and showed a reduction in their intracellular lysosomal level (de las Heras-García and Pampliega, 2022). It is possible that glial cells can be involved in these cell–cell interactions, adding another layer of complexity to the tripartite communication. It is yet unclear how astrocytes sense the absence of cilia in neurons. Astrocytes are ciliated but are inclined to become nonciliated in aged mouse brains (de las Heras-García and Pampliega, 2022). The exact roles of astrocytic cilia in the brain remain undefined. All these findings indicate the complexity of the neural tissue and the likelihood that cilia could function as a novel intercellular lever for communication between brain cells. In a speculative manner, the cilium might also function as a gauge for brain health and age and potentially as an early neuropathological sign of neuronal dysfunction.

Recent electron microscopy datasets have revealed that cilia in the brain are structurally diverse both within a given cell type and between different cell types. For example, astrocytic cilia are most frequently embedded in pockets, whereas neurons more commonly exhibit surface cilia. They also show diverse interactions with the cortical connectome (Ott et al., 2023; Wu et al., 2023). These differences suggest functionally distinct roles that call for extensive defining of proteomes of distinct neural and glial cell types. iBioID will be key to dissecting the molecular 722 diversity within cilia across cell types of the brain. It will also be crucial to define the protein composition of brain cilia in ciliopathies and neurological disorders, which is expected to unveil ciliary perturbations and their effects on neural signaling and circuits.

Our iBioID approach targeting neuronal cilia represents a successful adaptation of iBioID to characterize low-abundance cellular structures and organelles, such as the primary cilium, wherein only one is present on each neuron. This contrasts with synaptic compartments, which are present in thousands per neuron. We deployed a bait that localizes to the ciliary membrane to capture ciliary signaling pathways in the mature brain. Our focus was to address a broad interest in identifying the underexplored neural signaling that are mediated by the neural cilium. This approach mostly captured membrane-related proteins and did not systemically cover other ciliary compartments, including the ciliary barrier and the transition zone. Key ciliary cargoes, such as IFT and BBS complexes, were also not detected. A holistic strategy is therefore needed that combines several baits to achieve full coverage of the protein content of the neuronal cilium. Although cilia iBioID did not comprehensively cover every ciliary subcompartment, we identified novel neural-specific proteins with intriguing ciliary localization patterns that might be of relevance to brain architecture and function. In most cases, we noticed discrete foci along the cilium, sometimes with similar intensity to nonciliary localization.

Our finding is a proof-of-principle that further screening of the neuronal ciliome will be feasible in the brain. Our cilia iBioID will allow future studies to identify the molecular dysfunctions within neuronal cilia found in ciliopathies and neurodegenerative disorders. These future directions will bring greater understanding about cilia pathogenicity and how it could affect neural function and, in turn, lead to neurological deficits. This research lays the foundation for understanding the intrinsic signaling transmitted by the neuronal cilium, as well as potential external stimuli and downstream signals that influence brain function and homeostasis. The long-term goal would be to develop unique foundations for cilia biology in the brain by connecting novel neural ciliary signaling to subtypes of neurons and their specialized circuits, ultimately using the neuronal cilium as a targetable molecular lever in a novel therapeutic strategy to help correct neurological deficits in human disease.

### Limitations of the study

The ciliary baits used in this study are GPCRs, which are membrane-bound proteins that limit a comprehensive mapping of ciliary compartments. While we detected native biotin in all our conditions, it is still plausible that minimal nonciliary biotinylation might occur with our ciliary baits, potentially leading to false positives. Therefore, all remaining untested hits will require further validation, both in vitro and in vivo. Our study focuses on neuronal cilia without distinguishing between neuronal subtypes and does not include deeper brain regions, such as the striatum. The use of AAVs was limiting in this regard, and additional studies will need to account for neuronal diversity and target labeling across brain regions. Employing conditional mouse models could be a solution to overcome this limitation.
