# Supplementary figures and images for: Identification of New Ciliary Signaling Pathways in the Brain and Insights into Neurological Disorders
Source: J Neurosci. 2025 Jul 7;45(33):e0800242025. doi: 10.1523/JNEUROSCI.0800-24.2025 (PMC12352538; doi:10.1523/JNEUROSCI.0800-24.2025)

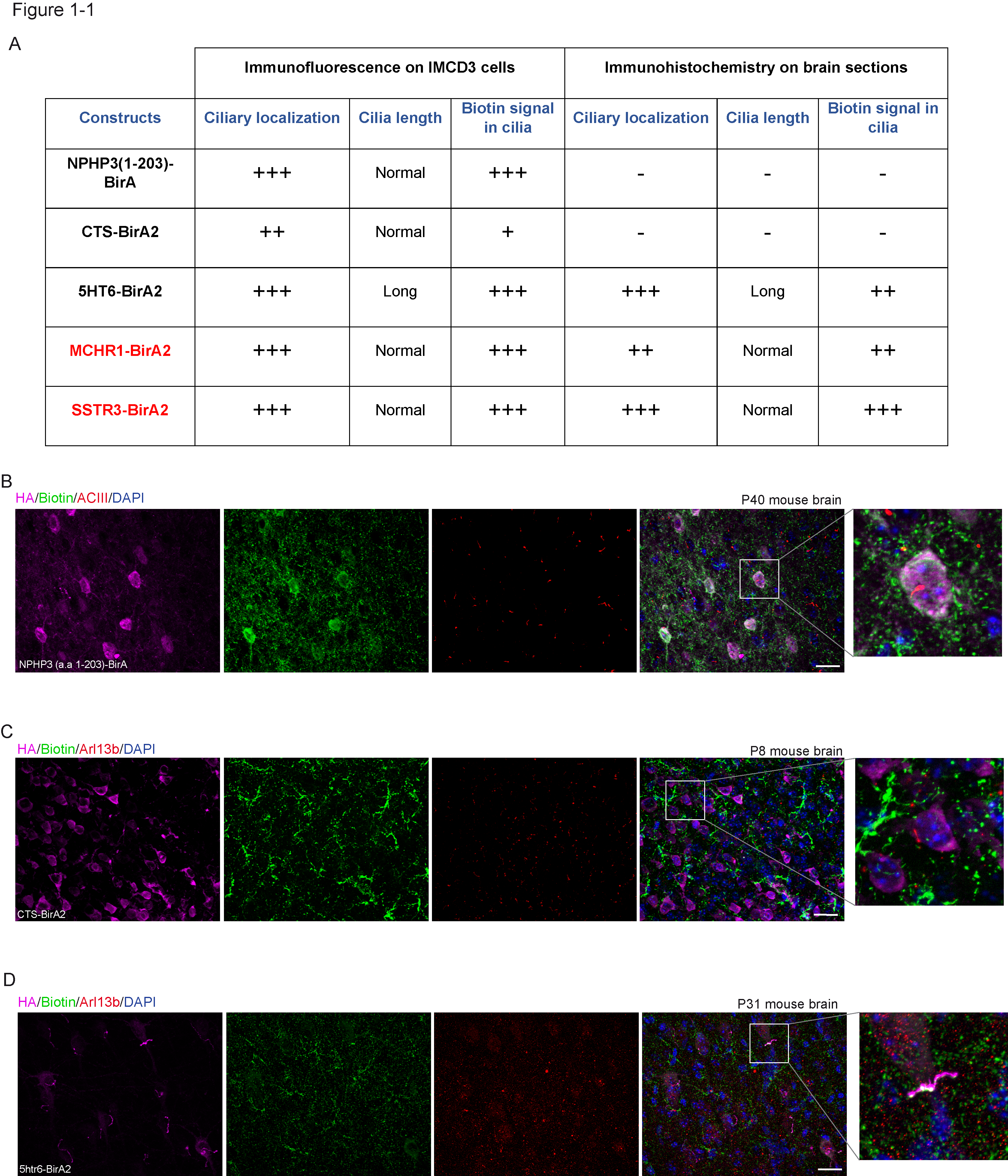

Supplement: Figure 1-1 — Testing of ciliary baits to target biotin ligaseA. A summary of tested ciliary baits to target the biotin ligase to neuronal cilia in vivo. Baits were first tested in IMCD3 cells and then assessed in the mouse brain, as detailed in figure 1A. Immunohistochemical labeling from mouse brain sections transduced withB. AAV-NPHP3 (a.a 1-203)-BirA.C. AAV- ciliary targeting sequence of Fibrocystin (CTS)-BirA2.D. AAV-5HTR6-BirA2. Sections are labeled with antibodies to Biotin (green), ciliary markers ACIII and Arl13b (red), and HA (magenta). DNA was stained with DAPI. Scale bars: 20 µm. Download Figure 1-1, TIF file. [file jneuro-45-e0800242025-s001.tif]

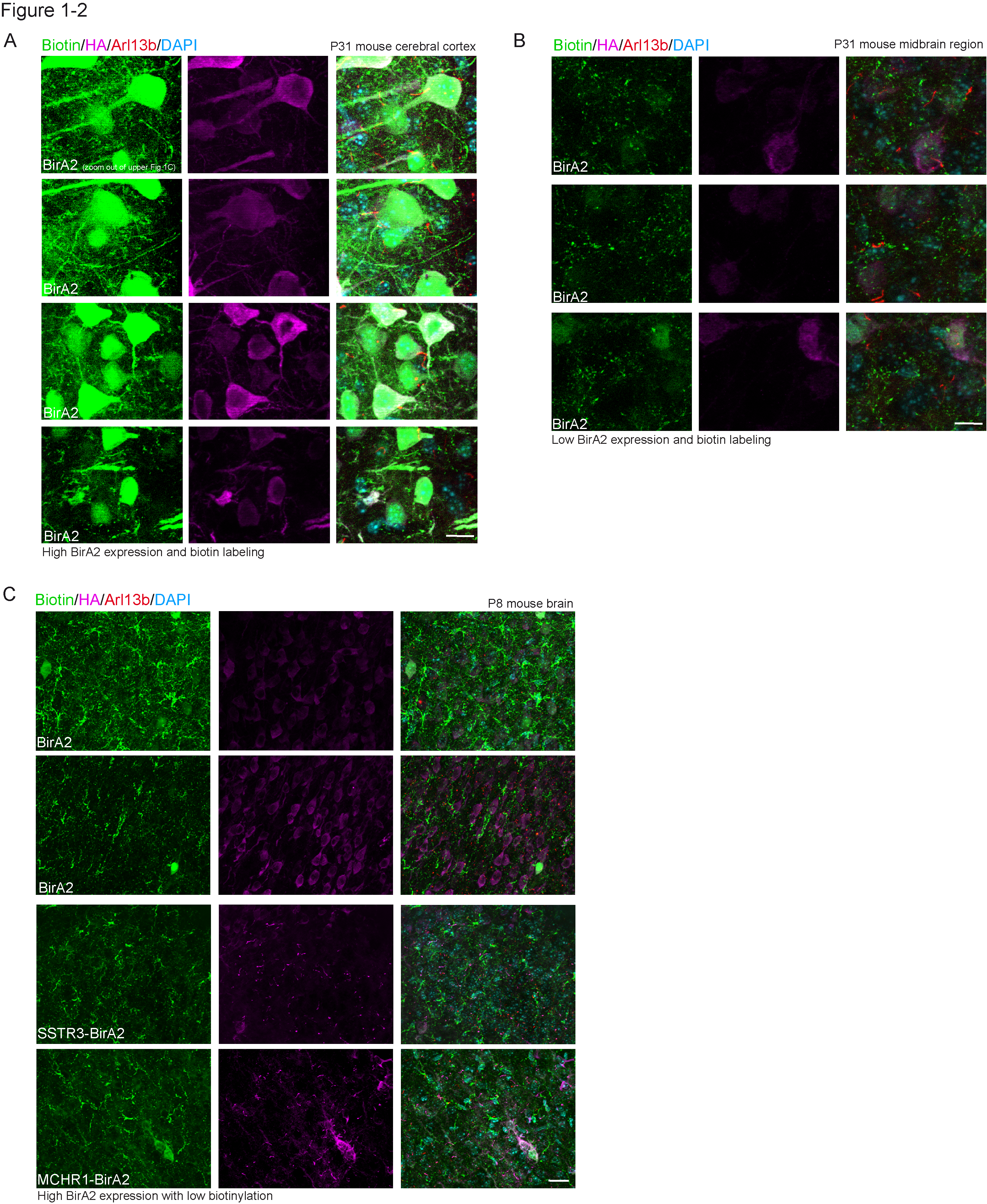

Supplement: Figure 1-2 — Native biotin labeling in BirA2 and GPCRs-BirA2 brainsA. ­Immunohistochemistry of P31 cortical slices transduced with AAV-BirA2 and labeled with antibodies to HA (fused to BirA2, magenta), Biotin (green), and Arl13b (red). DNA was stained with DAPI. Scale bars: 10 µm. Images were adjusted to approximately match the intensity range of the GPCR-BirA2 images (Figure 1C). Panels show high BirA2 expression and biotin labeling.B. same as A, with low BirA2 expression and biotin staining in the midbrain area.C. ­Immunohistochemistry of P8 brain sections transduced with AAV-BirA2 or GPCRs-BirA2 and labeled with antibodies to HA (fused to BirA2, magenta), biotin (green), and Arl13b (red). DNA was stained with DAPI. Scale bar: 20 µm. Images show high BirA2 expression with low biotinylation. Download Figure 1-2, TIF file. [file jneuro-45-e0800242025-s002.tif]

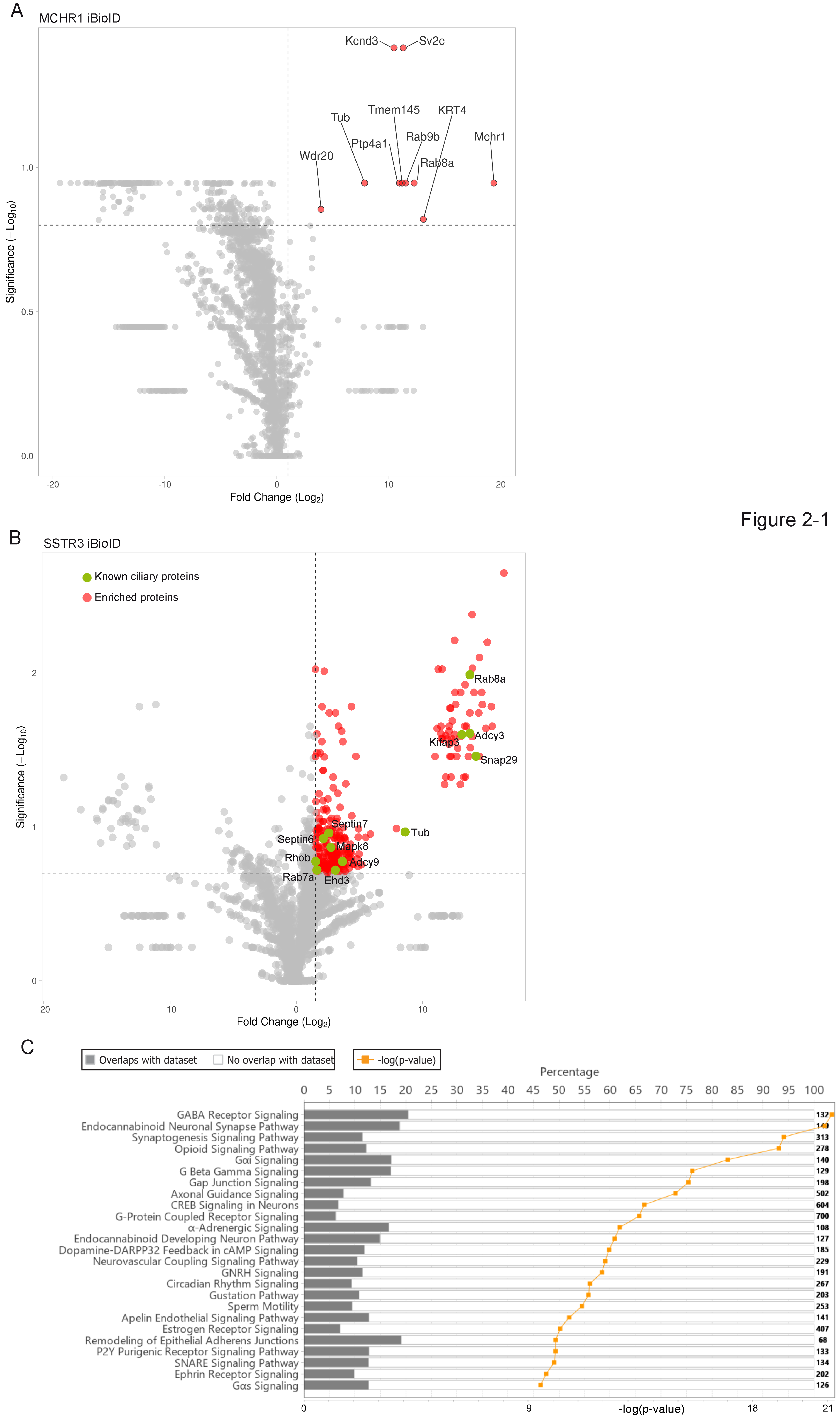

Supplement: Figure 2-1 — Volcano plots of SSTR3 and MCHR1 BioID and GO analysisA. Volcano plot of MCHR1-proximate proteins labeled by BioID. The red dots show the proteins considered hits, delimited by the selected thresholds: log2 fold change ≥ 2 and significance ≥ 0.7. The X-axis denotes the log2 fold change of MCHR1-BirA2: BirA2 control. In the Y-axis, significance displays the negative log10 transformed p-value for each protein.B. Volcano plot of SSTR3-proximate proteins labeled by BioID as shown in figure 2A. The green dots highlight known ciliary proteins overlapping with cilia proteomes discovered in Gupta et al., 2015 and Mick et al., 2015.C. Gene ontology analysis of hits enriched in SSTR3 iBioID. The table summarizes the most enriched signaling pathways with their corresponding p-values. Download Figure 2-1, TIF file. [file jneuro-45-e0800242025-s003.tif]

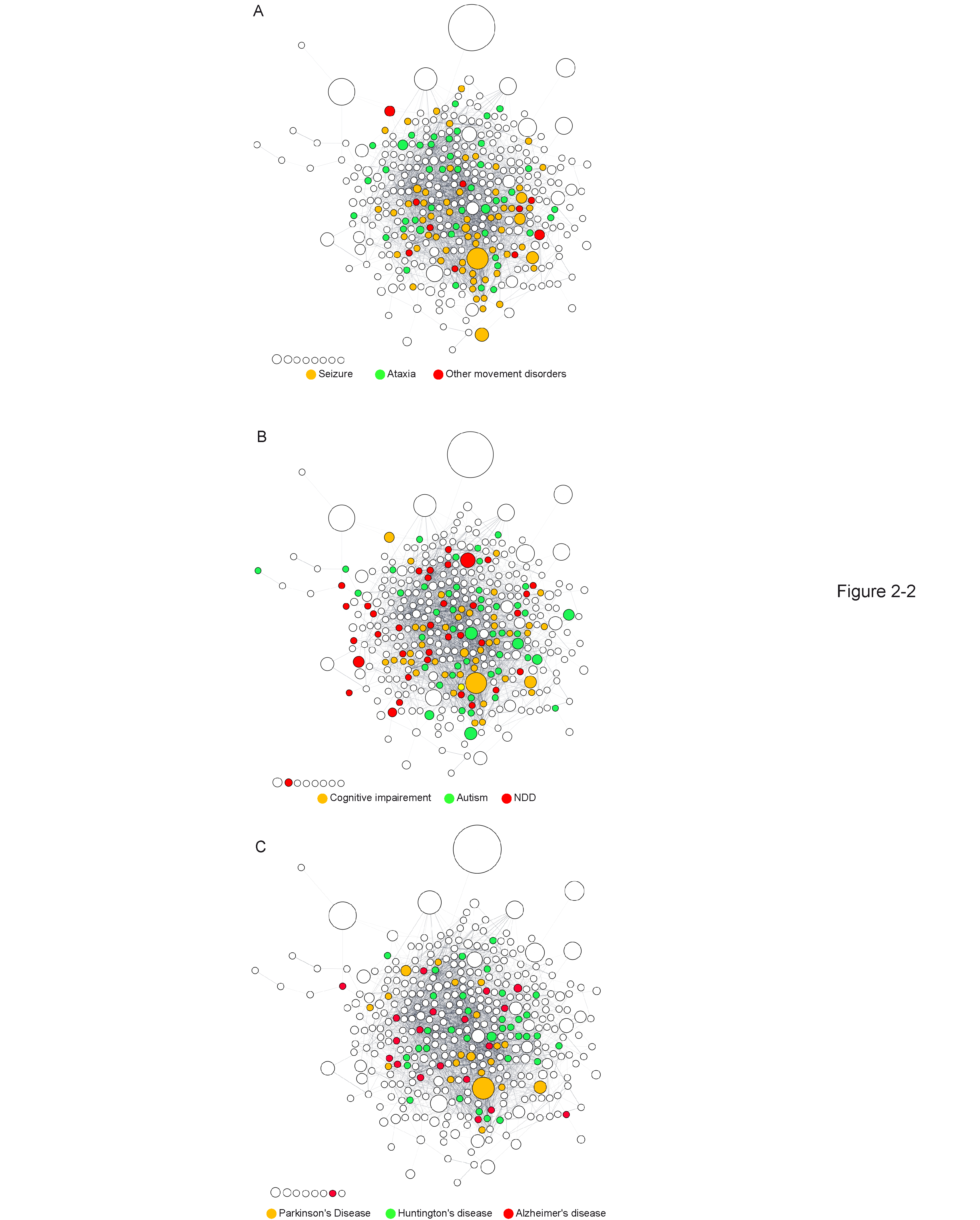

Supplement: Figure 2-2 — Insights into connections between neuronal cilia and brain disordersA. Clustergram topology of hits associated with cognitive impairment (orange), autism (green), and neurodevelopmental disorders (NDD, red)B. Clustergram topology of proteins associated with seizure (orange), ataxia (green), and other movement disorders (red)C. Clustergram topology of hits associated with Parkinson’s disease (orange), Huntington’s disease (green), and Alzheimer’s disease (red). For all clustergrams, overlapping hits were shown in one of the categories. Download Figure 2-2, TIF file. [file jneuro-45-e0800242025-s004.tif]

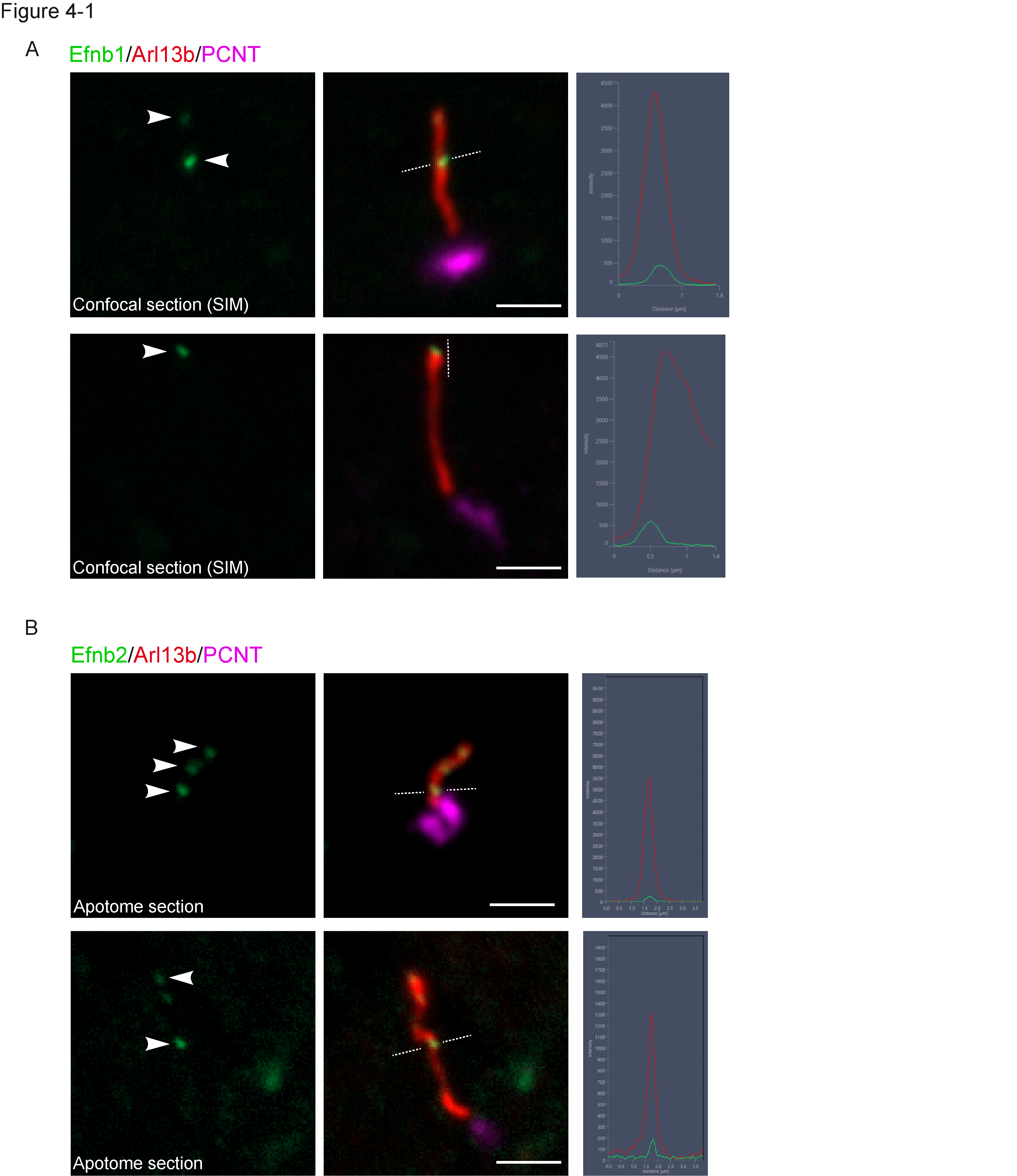

Supplement: Figure 4-1 — Efnb1 and Efnb2 are present in neuronal cilia Representative immunofluorescence confocal or apotome images of A. Efnb1 and B. Efnb2 (green) in neuronal primary cilia labeled with the ciliary marker Arl13b (red) and centrosomal Pericentrin (magenta). Arrows indicate the colocalization of Ephrins with Arl13b. Dashed lines indicate the selections used to generate fluorescence spectra graphs (Graphs showing intensity (A.U.) by distance (µm)), illustrating the intensity profiles of Efnb1 or Efnb2 (green) vs. Arl13b (red). Scale bars: 1 µm. Download Figure 4-1, TIF file. [file jneuro-45-e0800242025-s005.tif]
